# Optimization, Characterization, and Selection of Iron Ores as Oxygen Carriers for Application in Chemical Looping Processes

**DOI:** 10.1021/acsomega.5c05641

**Published:** 2026-01-22

**Authors:** Gineide Conceição dos Anjos, Tiago Roberto da Costa, Rebecca Araújo Barros do Nascimento Santiago, Gislane Pinho de Oliveira, Tomaz Rodrigues de Araújo, Rodolfo Luiz Bezerra de Araújo Medeiros, Ângelo Anderson da Silva de Oliveira, Dulce M. A. Melo, Renata Martins Braga

**Affiliations:** † Environmental Technology Laboratory (LabTam), 28123 Federal University of Rio Grande do Norte, 59078-970 Natal, RN, Brazil; ‡ Federal Institute of Rio Grande do Norte − IFRN, Campus Currais Novos, 59380-000 Natal, Brazil; § Coordination of Environmental Engineering, 37892 Federal University of Maranhão, MA-140, km 04, 65800-000 Balsas, MA, Brazil; ∥ Postgraduate Program in Chemistry, Federal University of Rio Grande do Norte, 59078-970 Natal, RN, Brazil; ⊥ Agricultural School of Jundiaí, Federal University of Rio Grande do Norte, 59078-970 Natal, RN, Brazil

## Abstract

The industrial viability of chemical looping technology is directly linked to the development of oxygen carriers (OCs) that meet the operational requirements of the process. This study investigates the optimization, characterization, and selection of iron ores from different regions of Brazil as potential OCs for chemical looping applications. A total of 13 samples were analyzed, including 11 predominantly composed of hematite and 2 of ilmenite. These materials were characterized through physicochemical, morphological, and structural analyses using techniques such as X-ray diffraction (XRD), X-ray fluorescence (XRF), temperature-programmed reduction (TPR), and scanning electron microscopy–energy-dispersive spectroscopy (SEM-EDS). The samples exhibited good mechanical strength (≥2.2 N), oxygen transport capacity ranging from 1.21% to 4.90%, and high reactivity during redox cycles with methane and hydrogen. Notably, the FeHP, FeHJ, FeHC, FeLC, FeTiHL, and FeTiHM samples demonstrated outstanding performance in terms of reactivity, cyclic stability, and oxygen transport capacity, showing suitability for operation in the typical temperature range of 800–1100 °C for CL processes. These findings highlight the potential of applying the selected materials in chemical looping technologies, offering sustainable and cost-effective alternatives for CO_2_ capture and utilization.

## Graphical abstract

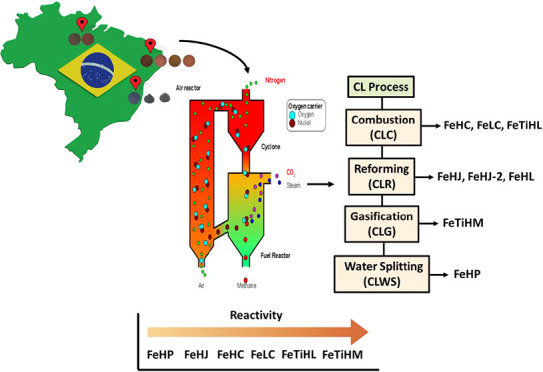

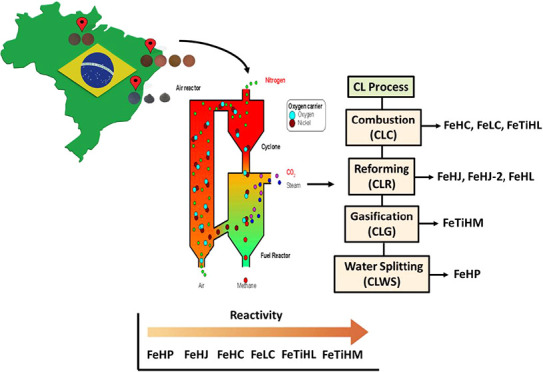

## 1 Introduction

Chemical looping (CL) technologies stand out for their environmental, energy, and economic advantages, enabling the efficient capture of CO_2_ generated during the production of heat, electricity, syngas, or hydrogen. This approach offers lower operational costs and reduced energy losses compared to conventional fuel combustion processes.^1^

The fundamental principle of CL processes is based on the indirect transfer of oxygen from air between two fluidized bed reactors—the air reactor (AR) and fuel reactor (FR)—to react with the fuel via an oxygen carrier (OC). In this system, the OC, a metal oxide (Me*_X_* O*_Y_*), facilitates oxygen transport between the reactors, eliminating direct contact between fuel and air.^2–4^ The most commonly adopted configuration consists of two interconnected fluidized bed reactors (Figure 1): the AR and FR, where the OC circulates continuously through a loop seal.^3,4^ Within FR, OC undergoes a reduction reaction, transitioning into its metallic form (Me) or a partially reduced state (Me_*x*_O_*y*‑*z*_), thereby oxidizing the fuel. Depending on process conditions, this reaction can be directed toward different objectives, such as energy generation or hydrogen production.^5^ The reduced OC is then transferred to the AR, where it is reoxidized upon exposure to oxygen from the air. These reduction–oxidation cycles are repeated continuously, ensuring a stable and efficient process. The overall energy balance of CL processes is comparable to that of conventional combustion technologies.

**Figure 1:**
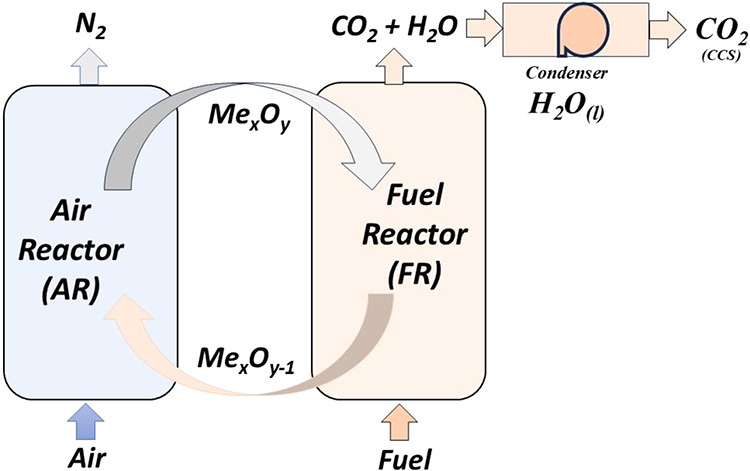
Schematic diagram of chemical looping (CL) processes using an oxygen carrier. Reprinted with permission from da Silva, A. A.; Melo, D. M. A.; da Costa, T. R.; Medeiros, R. L. B. A.; dos Anjos, G. C.; Carvalho, F. C.; Santiago, R. A. B. N.; Oliveira, Â. A. S.; Braga, R. M. Ni–Fe supported on CaAl2O4 obtained from egg shells for chemical looping technology. J. Energy Inst. 2025, 118, 101877. Copyright 2025 Elsevier.

One of the main advantages of CL is the absence of direct ai–fuel interaction in FR, which prevents the formation of NO*_x_* compounds.^1,6^ Additionally, the steam produced during the process can be easily separated by condensation, resulting in a concentrated CO_2_ stream (in combustion-based applications) that is readily available for transportation and storage.^7^

Chemical looping is a highly versatile technology, capable of using various fuel types (solid, liquid, and gas) to obtain different products, such as heat, electricity, syngas, or hydrogen. These outcomes can be achieved through different CL-based processes, such as combustion, reforming, or gasification.^2,8^ Furthermore, CL facilitates cost-effective carbon capture and storage (CCS), offering a more economical alternative to conventional CO_2_ capture processes, both first and next-generation technologies.^9^

Despite the advancements in the design of fluidized bed reactors and the development of pilot plants for CL processes, many of which have reached a technological maturity level (TRL) above 5 and are well established, the primary challenge remains the development of OC with properties suitable for large-scale industrial applications. For an OC to perform effectively under CL operating conditions, it must meet several criteria: be able to thermodynamically convert the fuel; have a high oxygen carrying capacity (*R*_OC_) and high reactivity with both oxygen and the fuel; minimize coke formation; have good fluidization properties and high resistance to friction and agglomeration; be environmentally friendly, low cost, and readily available in large quantities.^4,10–12^

The proper choice of the oxygen carrier (OC) is critical to achieving high conversion rates in the chemical looping (CL) process. In addition to providing oxygen for fuel oxidation, OCs can also act as catalysts in certain reduction reactions and facilitate heat transfer from the air reactor (AR) to the fuel reactor (FR). Studies indicate that nickel, cobalt, iron, copper, and manganese metal oxides are the most widely investigated candidates for CL application due to their favorable thermodynamic properties for converting CH_4_, H_2_, and CO under the CL process’s typical operating conditions.^10,13^ In particular, the redox pairs such as Fe_2_O_3_/Fe_3_O_4_, MnO_2_/Mn_2_O_3_, Mn_2_O_3_/Mn_3_O_4_, Co_3_O_4_/CoO, and CuO/Cu_2_O have demonstrated high efficiency in methane reactions. Nickel-and iron-based redox pairs exhibit optimized performance at elevated temperatures.^4^ From a thermodynamic perspective, materials such as CuO, Co_3_O_4_, Fe_2_O_3_, Cu_2_O, and CoO have strong oxidizing properties, allowing nearly complete fuel conversion.^14^

Despite these advantages, the literature points out that the high production costs of synthetic metal oxide OC pose a significant barrier to their application in industrial-scale processes. As an economical and viable alternative, natural ores and ore residues have been gaining prominence within the scientific community.^15^ The metal oxides present in these materials exhibit similar properties, in some cases superior, to those of synthetic OCs. Additionally, natural ores can serve as both active phases in redox reactions and as inherent natural support or additives to improve the physicochemical properties of the OCs. Beyond their favorable physicochemical characteristics, natural ores have several advantages, including low cost, widespread availability, and heterogeneous chemical composition, which promote synergistic effects between their constituent phases. Furthermore, they can be processed to optimize their performance.^4,12^ Iron, manganese, and copper-based ores are noteworthy due to their nontoxicity, low cost, and large-scale availability, making them more competitive compared to synthetic materials and nickel- or cobalt-based ores.^2^

Within this context, iron-based oxygen carriers (OCs) have been extensively studied and applied in various chemical looping (CL) technologies, particularly in combustion, gasification, and reforming processes. The key attributes that make iron-based materials attractive, besides their low cost, include good redox performance, wide availability, ease of procurement, high oxygen transport capacity,^16^ high sintering temperature, reduced coke formation, and strong resistance to the formation of sulfides and sulfates.^14,17–19^

Iron ores have been investigated in various chemical looping (CL) technologies, demonstrating promising results. In biomass gasification processes, they have been shown to enhance carbon conversion, promote high syngas formation, and reduce tar levels.^16,20^ Hematite, in particular, has been tested for extended periods in pilot-scale chemical looping combustion (CLC) plants, consistently exhibiting high reactivity with methane.^21^ In experiments with bituminous coal using *in situ* gasification chemical looping processes (iG-CLC), combustion efficiencies of up to 96% have been achieved.^22^

Another widely studied iron-based ore is ilmenite, composed mostly of FeO·FeTiO_2_ (Fe_2_TiO_3_). This material has been successfully employed as an OC, showing a progressive increase in oxygen transport capacity and reactivity throughout redox cycles, until reaching stability comparable to the Fe_2_O_3_ system.^23,24^ Ilmenite can achieve an *R*_OC_ of up to 5% with moderate methane conversion and acceptable performance in syngas production.^25^ However, the main disadvantage of ilmenite is the potential segregation of iron and titanium oxides during repeated redox cycles, which can hinder its complete regeneration and gradually reduce its oxygen transport capacity over time.^24^ Despite this, the experimental *R*_OC_ values remain sufficiently high to ensure high fuel conversion. Precalcination of ilmenite at temperatures between 900 and 1200 °C has been shown to enhance its activation and reactivity with H_2_, CO, and CH_4_, although it can also be used in its natural state.^25^ A comprehensive review of ilmenite properties and applications is available in the review article of Bartocci et al.^26^

Several high-impact review articles have explored topics closely related to this study, including CO_2_ capture by CL processes,^2,11,27,28^ the development of oxygen carriers, as well as the application of ores as OCs in different CL technologies,^4,12^ and iron-based ores applied in CL technologies.^16,29^

Given that iron-based ores exhibit significant potential as OC, their use can accelerate the advancement of CL technologies toward large-scale commercial applications, overcoming both technological and economic challenges. In this scenario, Brazil is poised to play a pivotal role, as it is one of the world’s largest producers of iron ore. Based on this, this study investigates 13 different iron ore samples from different regions of Brazil, 11 of which are composed mostly of iron oxides (hematite) and two are ilmenite-type compound oxides, as potential oxygen carriers. Due to Brazil’s vast territorial extent, these materials have different chemical compositions and were formed under distinct geological conditions, which significantly influences their physicochemical properties. Thus, this work aims to conduct a detailed analysis of the morphological, structural, and physicochemical properties of these ores and assess how these properties affect their reactivity and oxygen transport capacity during redox cycles. A comprehensive understanding of these factors is essential to evaluate their impact on material reactivity and thus propose strategies to overcome OC manufacturing limitations, ultimately facilitating their industrial-scale application.

## 2 Materials and Methods

### 2.1 Preparation for the Oxygen Carrier

Thirteen iron ore samples were selected, optimized, characterized, and investigated as potential oxygen carriers (OCs). Among these, 11 samples primarily consisted of hematite, while 2 were mainly composed of ilmenite as the active phase. The samples were collected from various Brazilian states, including Rio Grande do Norte, Bahia, Paraba, Pará, and Minas Gerais. Table 1 presents the nomenclature adopted for each sample based on the content of the active phase and its source location.

**Table 1:**
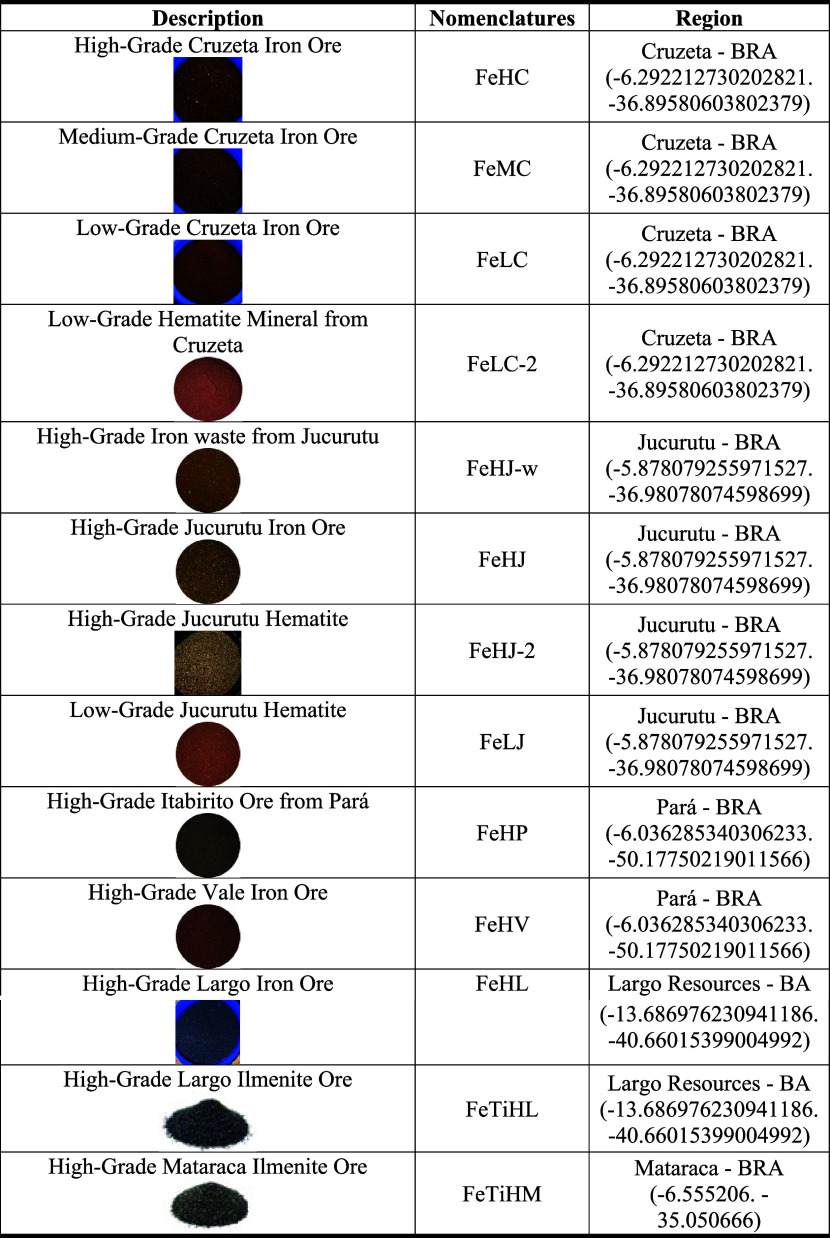
Description of the Iron Ores and Nomenclatures Adopted.

The raw ore samples went through a grinding and sieving process to optimize their granulometry, in order to obtain an average particle diameter size between 100 and 300 μm. FeTiHL showed a particle diameter of less than 100 μm. To increase its particle size, the sample was submitted to a granulation stage employing an intensive EL5 Profi-Erich mixer. Corn starch was used as a binder agent. As a means to remove the starch, the granulated mixture was heated from room temperature up to 900 °C, at a 10 °C·min^–1^ rate and then held at 900 °C for 3 h. After this procedure, FeTiHL particles were also ground and sieved to obtain a particle size between 100 and 300 μm.

### 2.2 Oxygen Carrier Physicochemical Characterization

The optimized ores were submitted to several characterization techniques in order to investigate their structural, morphological, and physicochemical properties.

The semiquantitative chemical composition was determined by X-ray fluorescence (XRF), using Shimadzu EDX 720 equipment, with a rhodium anode (Rh), voltage of 50 kV, and Si/Li detector. The results were presented in the form of oxides. The particle size distribution was analyzed by using a CILAS 920L laser granulometer, employing dry methodology. The helium gas pycnometry technique was employed to determine the specific mass of the materials using AccuPyc 1340 equipment from Micromeritics. The measurements were conducted in a cell with a capacity of 11.80 cm^3^, with ten readings for accuracy.

The OC’s crystalline phases were investigated by X-ray diffraction (XRD) in a Shimadzu XRD-7000 diffractometer, with Cu Kα radiation (λ = 1.5409 Å), operating at 40 kV and 30 mA. The diffractograms were obtained in the 2θ range from 10° to 80°, with a scanning speed of 1.0°·min^–1^ and a step of 0.02°. The identification of the crystalline phases was carried out based on the standards of the Joint Committee on Powder Diffraction Standards (JCPDS).

The surface morphological characteristics of the OC in nature were analyzed by scanning electron microscopy (SEM), using the VEGA TESCAN equipment. To prepare the samples, their surfaces were metallized with a thin layer of gold by the sputtering method, using a current of 10 mA for 60 s. This procedure was performed to confer the electrical conductivity required for obtaining high-quality images. The semiquantitative chemical composition was determined by energy-dispersive spectroscopy (EDS), using the EDS 30 mm^2^ detector coupled to the SEM, with an operating voltage of 20 keV.

The reduction profiles were determined by temperature-programmed reduction (TPR) using Micromeritics’ Autochem II equipment. The analysis was conducted in a U-shaped fixed-bed quartz reactor coupled to a heating furnace. A 100 mg sample was subjected to a heating rate of 10 °C·min^–1^, from room temperature to 800 °C, under a gas mixture composed of hydrogen (10%) and argon (90%), with a flow rate of 50 mL·min^–1^. Hydrogen consumption was monitored by a thermal conductivity detector (TCD).

### 2.3 Oxygen Carrier Preselection

Ore’s preselection was based on their crushing strength (>1 N), high hematite or ilmenite phase concentration, and high H_2_ consumption during TPR experiments. Based on these criteria, FeHC, FeLC, FeLC-2, FeHJ, FeHJ-2, FeHP, FeHL, FeTiHL, and FeTiHM ores were selected for thermobalance reactivity evaluation under different experimental conditions. Figure 2 presents a detailed flowchart of the experimental procedure used in the selection of ores with promising properties for application as oxygen carriers.

**Figure 2:**
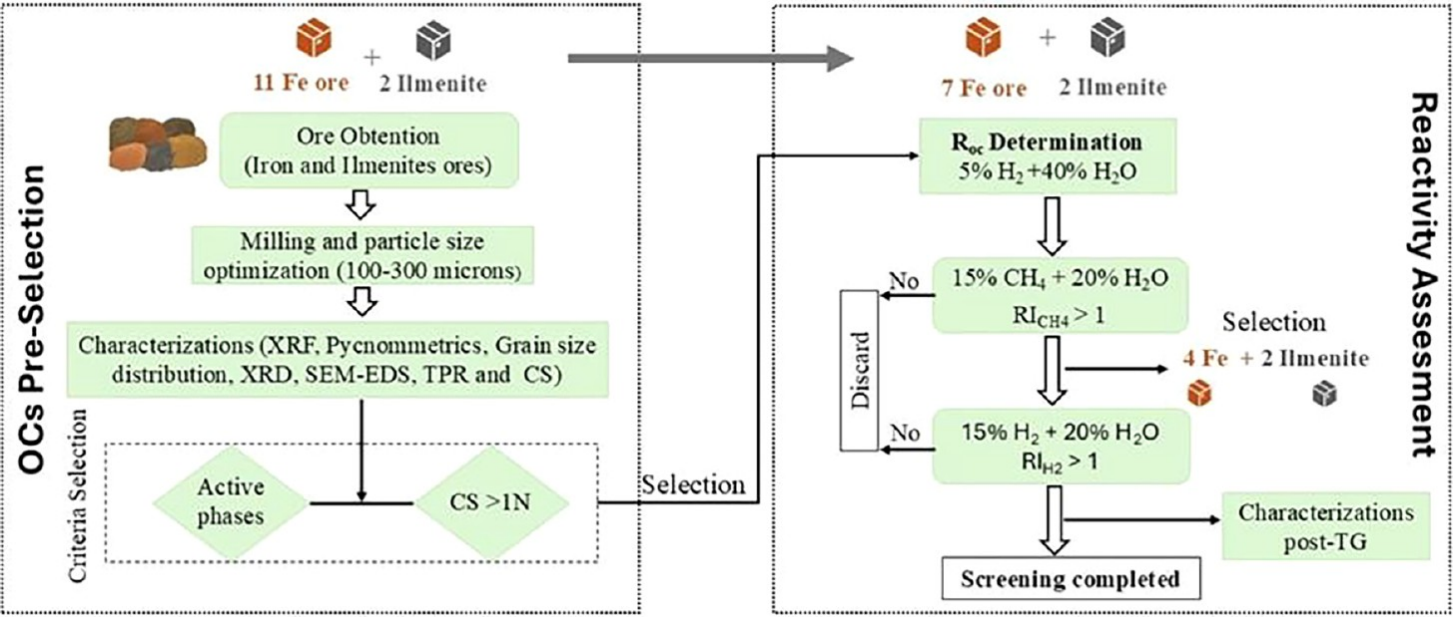
Flowchart of the methodology used to select materials for evaluation as oxygen carriers.

### 2.4 Oxygen Carrier Reactivity Characterization

The redox reactivities and oxygen transport capacities of the preselected ores were assessed by conducting experiments on an adapted thermobalance, model CI Electronics. The thermobalance is equipped to operate with different mixtures of reactive gases (H_2_, CH_4_, and Synthetic Air). Figure 3 presents a schematic diagram of the thermobalance equipment.

**Figure 3:**
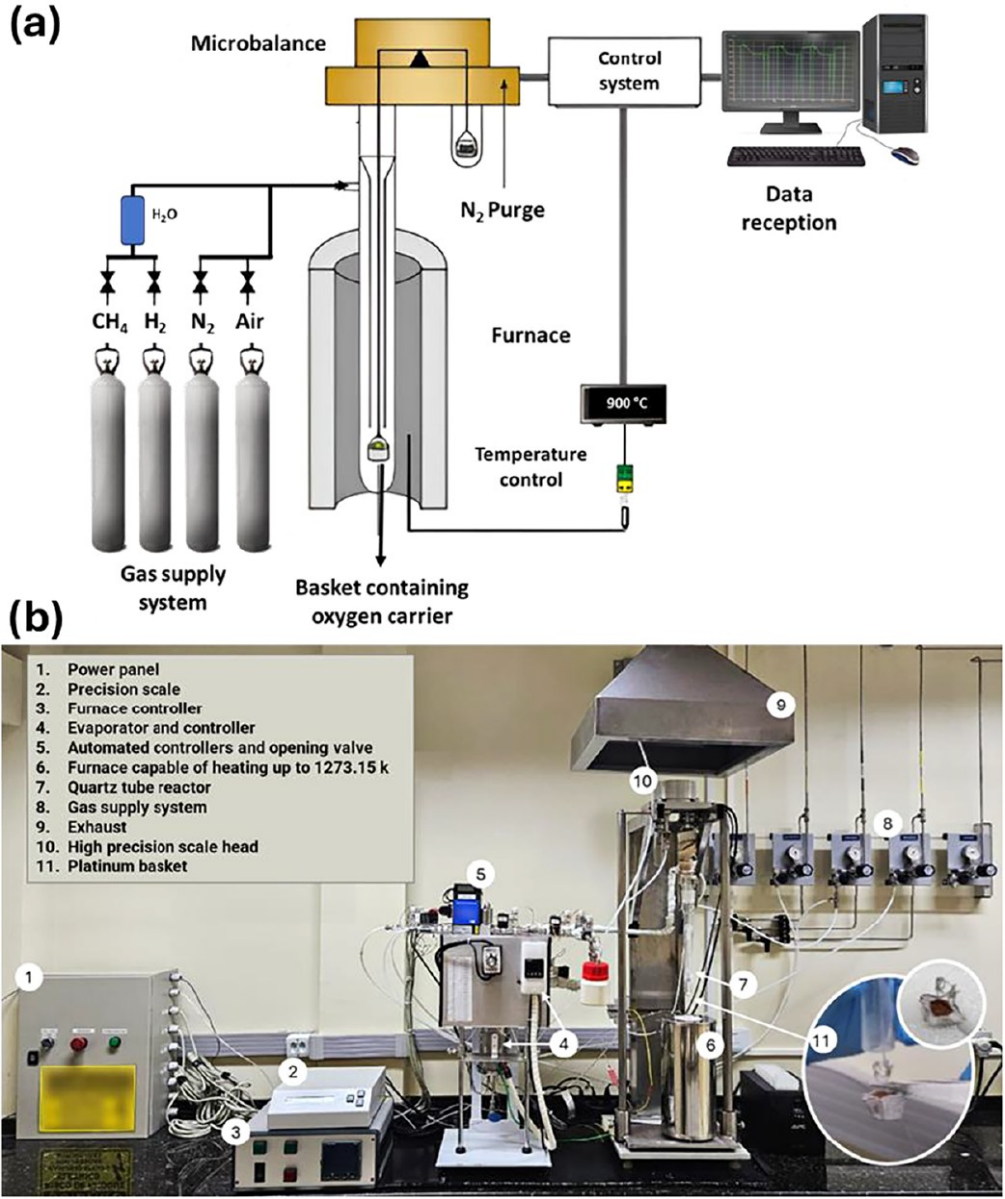
High-precision thermobalance adapted for reactivity analysis in reduction/oxidation cycles: (a) schematic diagram and (b) equipment used.

The redox cycle experiments in the thermobalance were carried out using about 50 mg of the ore sample, which was positioned in a platinum mesh basket (item 11 of Figure 3). This basket was suspended by rods connected to the high-precision scale (item 10) and inserted into a quartz tube reactor (item 7). Initially, the sample was heated in an air atmosphere by a furnace surrounding the reactor (item 6), at a 20 °C·min^–1^ heating rate, until it reached the experimental temperature of 900 °C. The gas total flow was 25 L_N_·h^–1^, controlled by an automatic system composed of automatic valves and flow controllers (item 5). Upon reaching the operating temperature and stabilizing the system, the sample was exposed to alternating reduction and oxidation conditions, as described in Table 2.

**Table 2:** Detailed Description of the Purge, Reducing, and Oxidizing Mixture Composition, and Reactivity Experimental Conditions Performed in the Thermobalance.

	reducing mixture	*T*_evap_ ^c^ (°C)	^b^ *t*_red_	oxidizing	^c^ *t*_oxi_	^e^ *T*_reator_ (°C)
I	5% H_2_ + 40% H_2_O	75	máx 30 min	100% Ar	máx 30 min	900
II	15% CH_4_ + 20% H_2_O	60	máx 30 min	100% Ar	máx 30 min	900
III	15% H_2_ + 20% H_2_O	60	máx 30 min	100% Ar	máx 30 min	900
IV	15% H_2_	0	máx 30 min	100% Ar	máx 30 min	900

	purge	*T*_evap_ ^a^(°C)	^d^ *t*_purge_	*T*_reactor_ (°C)		
V	100% N_2_	0	2 min	900		

a *T*_evap_: evaporator temperature.

b *t*_red_: reduction time.

c *t*_oxi_: oxidation time.

d *t*_purga_: purge time.

e *T*_reator_: reactor temperature. N_2_ was used to balance the reducing mixture.

Each redox step was conducted until the sample mass remained stable, ensuring that no single reduction or oxidation step exceeded 30 min. In all instances, synthetic air served as the oxidizing agent. Nitrogen gas was used to balance the reducing mixture and to purge the system between the reduction and oxidation steps for 2 min, thereby preventing any unwanted mixing of reactive gases. Water vapor, a component of the reducing mixture, was generated by an evaporator (item 4) equipped with a temperature control. The evaporator was initially filled with 120 mL of distilled water. The percentage of water vapor (or vapor pressure) in the mixture was regulated by the temperature within the evaporator, achieving 40% steam at 60 °C and 20% steam at 75 °C.

The mass variation over time was recorded during three complete reduction and oxidation cycles, as illustrated in Figure 4a, using LabWeight software. This number of cycles was considered sufficient for initial screening and comparative evaluation of the different ores, as previous studies have shown that iron-based carriers, especially natural ores, can exhibit clear activation or deactivation trends within the initial cycles, allowing for a robust preliminary assessment of their cyclic stability and performance potential. Data from the last redox cycle (Figure 4b) for each reducing gas mixture was used to determine the oxygen carrying capacity (*R*_oc_), solids conversion during reduction (*X*_red_) and oxidation (*X*_oxi_), and rate index (RI_TGA_).

**Figure 4:**
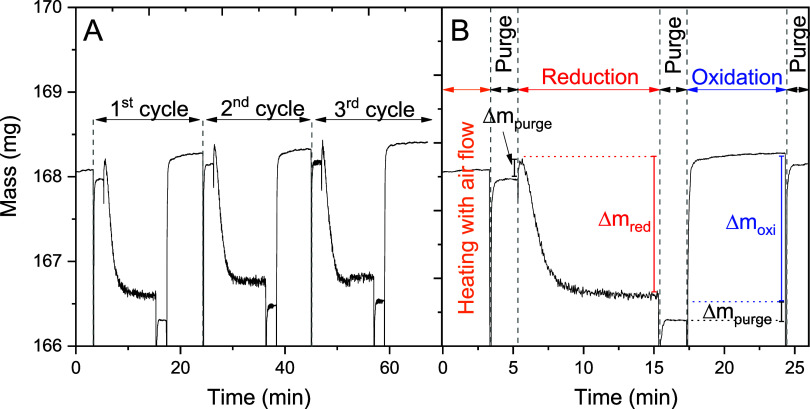
Mass data acquisition over time (a) for three redox cycles and (b) for a single redox cycle.

During the reduction step, the OC loses mass by delivering oxygen from its crystal structure to oxidize the reducing gas mixture, as illustrated in Figure 4b.

The compositions of the reducing mixtures were strategically selected to thermodynamically control the degree of reduction of iron oxides, allowing the assessment of the OC performance under different process objectives. The partial pressure of water vapor (H_2_O) plays a critical role in determining the thermodynamic equilibrium of the reduction reactions. The mixture with 5% H_2_ + 40% H_2_O (H_2_O/H_2_ ratio = 8.0) limits the reduction primarily to the Fe_2_O_3_/Fe_3_O_4_ system, simulating conditions for complete combustion (CLC), where the oxygen carrier is only partially reduced to maintain high reactivity and oxygen transport capacity. In contrast, the mixtures with lower water vapor content of 15% CH_4_ + 20% H_2_O (H_2_O/CH_4_ ratio = 1.33) and 15% H_2_ + 20% H_2_O (H_2_O/H_2_ ratio = 1.33) allow for deeper reduction steps. Specifically, the H_2_O/H_2_ ratio of 1.33 is below the thermodynamic equilibrium limit of approximately 2.28 required for the FeO → Fe transition at 900 °C.^30^ This ensures that the reduction is thermodynamically controlled to stop predominantly at FeO, preventing full reduction to metallic iron under the experimental conditions employed. Such conditions are relevant for processes like syngas production and gasification (CLR/CLG), where deeper reduction enhances the oxygen carrier’s capacity to participate in reforming and partial oxidation reactions. By controlling the gas composition, we can simulate and evaluate the performance of the oxygen carriers across a range of operating conditions encountered in different chemical looping applications.

#### 2.4.1 Data Evaluation

The oxygen transport capacity parameter (ROC) represents the maximum amount of oxygen that can be transferred by the carrier during a complete reduction–oxidation cycle, expressed as the weight percentage difference between the fully oxidized and reduced states of the material. ROC is calculated according to eq 1:

1 $$R_{\mathrm{oc}}=X_{\mathrm{TO}}\cdot R_{o}=X_{{\mathrm{Me}}_{x}O_{y}}\cdot \frac{m_{\mathrm{oxi}}-m_{\mathrm{red}}}{m_{\mathrm{oxi}}}$$

For iron-based oxygen carriers, the theoretical oxygen transport capacity (*R*_o_) varies depending on the redox system involved. For instance, the Fe_2_O_3_/Fe_3_O_4_ system has a theoretical *R*_o_ of 3.4 wt %, while Fe_2_O_3_/FeO and Fe_2_O_3_/Fe^0^ systems can reach 10 and 30 wt %, respectively. In the case of ilmenite-based carriers, the Fe_2_TiO_5_/FeTiO_3_ system exhibits a theoretical *R*_o_ of 5.0 wt %, and the FeTiO_3_/Fe^0^ + TiO_2_ system can theoretically reach 31.6 wt %.^29^ However, the experimental ROC values obtained for each material depend on the thermodynamic conditions imposed by the reaction medium, particularly the presence of water vapor in the reducing gas and the actual degree of reduction achieved by the oxygen carrier during operation.

The conversions during the reduction (*X*_red_) and oxidation (*X*_oxi_) steps, highlighted in Figure 3b, represent the fraction of oxygen supplied by the OC, and the fraction of oxygen recovered relative to its maximum capacity (*R*_oc_), respectively. These conversions are calculated according to eqs 2 and 3.^31^

2 $$X_{\mathrm{red}}=\frac{m_{o}-m}{m_{o}-m_{r}}=\frac{m_{o}-m}{R_{\mathrm{OC}}m_{o}}$$

3 $$X_{\mathrm{oxi}}=\frac{m-m_{r}}{m_{o}-m_{r}}=\frac{m-m_{r}}{R_{\mathrm{OC}}m_{o}}$$

The rate index, RI_TGA_ [%·min^–1^], is a parameter designed to compare the reactivities of different materials. It is calculated according to eq 4 and takes into account both the OC’s ability to transfer oxygen and the rate at which it is able to deliver it. This characteristic is represented by the slope of the conversion curve over time

$\left(\frac{dx}{dt}\right)$ , measured during the first minute of the experiment.^32^

4 $${\mathrm{RI}}_{\mathrm{TGA}}=100\times 60\times R_{\mathrm{OC}}\times \frac{p_{\mathrm{ref}}}{p_{\mathrm{TGA}}}\times \frac{dx}{dt}$$

where *p*_ref_ is the reference pressure, assumed to be 0.15 atm for the reduction stage and 0.1 atm for the oxidation stage, and *p*_TGA_ is the partial pressure of the reducing or oxidizing gas used in the thermobalance experiments.

### 2.5 OC Evaluation after the Reactive Process in Thermobalance

X-ray diffraction analyses were conducted for the reduced samples (after TPR analysis) and for the oxidized samples (after redox cycles performed in the thermobalance), aiming to identify and evaluate the structures formed during these reactive processes, providing a more detailed understanding of phase transitions for the investigated materials. The parameters and equipment used were the same as those previously described in item 2.2.

## 3 Results and Discussion

### 3.1 Characterization and Selection of Iron-Based Materials

In this section, the main results of the structural, physicochemical, and morphological characterizations to which the iron-based ores were submitted are presented in Table 3.

**Table 3:** Specific Mass, Average Particle Diameter, Crushing Strength, and Chemical Composition of Iron Ore Samples.

				chemical composition (%)^a^						
sample	density (g/cm^3^)	average diameter (μm)	crushing strength	Fe_2_O_3_	SiO_2_	Al_2_O_3_	MnO	CaO	TiO_2_	other
FeHC	3.99	262.12	3.08 ± 0.84	80.35	18.38		0.48	0.29		0.50
FeMC	3.36	248.35	3.07 ± 0.55	71.20	25.26		0.69	0.35	0.49	2.01
FeLC	3.32	203.54	3.64 ± 0.89	52.96	27.98	12.32	0.54	0.25	0.47	5.48
FeLC-2	2.76	150.37	2.55 ± 0.66	36.26	13.63	47.50	0.07		2.22	0.32
FeHJ-w	3.56	220.65	3.18 ± 0.90	84.60	14.08		0.56	0.11		0.65
FeHJ	3.65	287.48	3.40 ± 0.94	73.91	22.33		0.66	2.69		0.41
FeHJ-2	3.13	230.70	2.94 ± 0.77	70.63	27.98		1.38			
FeLJ	2.85	234.36	1.64 ± 0.56	54.67	39.72		0.56	0.48	1.07	3.5
FeHP	4.03	242.09	2.78 ± 0.66	90.46	6.33		3.03	0.18		
FeHV	4.69	292.76	2.66 ± 0.79	99.79			0.20			
FeHL	4.69	155.48	2.50 ± 0.71	91.25	3.75		0.32	0.48	3.87	0.42
FeTiHL	4.37	257.39	2.26 ± 1.04	64.84			1.05	0.31	33.73	0.07
FeTiHM	4.20	312.13	2.95 ± 0.64	50.46	2.96		1.69		43.99	0.9

a Measurement performed by XRF.

Table 3 shows that the ores primarily consist of iron oxides, with FeHP, FeHL, and FeHC ores containing more than 90% iron oxides. Other identified compounds include SiO_2_, Al_2_O_3_, K_2_O, CaO, and MgO. Silica (SiO_2_) and alumina (Al_2_O_3_) can act as supports for the active phases, while CaO, MgO, and K_2_O function as chemical additives, enhancing the chemical stability during successive redox cycles. These compounds have the potential to improve the performance of oxygen carriers. Alkali metals may boost OC reactivity and reduce coke formation.^4,33,34^ FeTiHL and FeTiHM samples have over 94% iron and titanium oxides, likely from the ilmenite phase (FeTiO_3_). These levels exceed those reported for Vietnamese^35^ and Chinese^15,36^ ilmenite. XRF quantifies simple oxides but cannot differentiate between mixed oxides. Additionally, the presence of low levels of MnO in some samples can increase the OC’s oxygen transport capacity by acting as an active phase.

It is noteworthy that the OC particles’ size and density affect the fluid dynamics of a continuous fluidized bed unit.^37^ Therefore, the average particle diameter has been optimized within the range of 100–300 μm to ensure adequate fluidization behavior in a circulating fluidized bed (CFB) reactor, minimize fines loss, and provide acceptable resistance to heat and mass transfer limitations, which is the recommended range for CL systems.^38^ The specific masses of most ores range from 3.1 to 4.7 g·cm^–3^, indicating satisfactory density. High-density particles can increase the minimum fluidization velocity and may cause agglomeration issues.^39^ All of the ores investigated, except FeLJ, exhibited crushing strength >2.2 N, which exceeds the recommended limit for CL fluidized beds (>1 N).^39^

Table 4 presents the crystal structures, unit cell types, and JPCDS and ICSD chart references obtained from diffractograms of the ores *in natura*. As indicated in Table 3, hematite (Fe_2_O_3_) was identified as the main active phase present in the ores analyzed. During the reduction stage of CL processes, iron oxide can assume different oxidation states (Fe_2_O_3_ → Fe_3_O_4_ → FeO → Fe) while delivering the oxygen necessary for fuel combustion. Thermodynamically restricting the reduction of hematite to magnetite (Fe_2_O_3_ → Fe_3_O_4_) is essential to the CLC process, as it promotes complete combustion, resulting in high-purity CO_2_. On the other hand, achieving the wustite (FeO) and metallic iron (Fe^0^) oxidation states is desirable in chemical looping reforming (CLR) and chemical looping gasification (CLG) processes, since this reduction leads to incomplete combustion, increasing CO and H_2_ levels.^1,9^ The FeHC, FeLC, FeLJ, FeHP, and FeHV samples also showed magnetite (Fe_3_O_4_) as the active phase. Wustite (FeO) was also identified in the FeLC-2 and FeHV samples. These ores, containing iron oxide phases in different oxidation states (Fe^3+^ and Fe^2+^), are oxidized to the iron phase in its highest oxidation state (Fe_2_O_3_) under oxidizing conditions during the initial stage of the reactivity test until reaching the reaction temperature. For iron-based OC, initiating redox reactions with hematite (Fe_2_O_3_) as the main active phase is advantageous, as it maximizes the theoretical oxygen transport capacity of these materials. The reactivity of these samples will be evaluated based on the reduction and oxidation cycle of the Fe_2_O_3_/Fe_3_O_4_ system.

**Table 4:** Parameters of the Crystalline Phases Found in the Iron Ore Samples.

sample	crystalline phase	JPCDS card	unit cell	ICSD
FeHC	Fe_2_O_3_	01–089–0597	trigonal	082135
	Fe_3_O_4_	01–086–1353	cubic	082447
	SiO_2_	01–089–8941	trigonal	089283
	SiO_2_	01–083–0540	trigonal	079635
FeMC	Fe_2_O_3_	01–089–0597	trigonal	082135
	SiO_2_	01–089–8935	trigonal	089277
FeLC	Fe_2_O_3_	01–089–0598	trigonal	082136
	Fe_3_O_4_	01–089–0950	cubic	085806
	SiO_2_	01–089–8936	trigonal	089278
FeLC-2	Fe_2_O_3_	01–089–0598	trigonal	082135
	Fe_2_TiO_5_	01–076–1743	orthorhombic	036183
	SiO_2_	01–078–1254	trigonal	062406
	SiO_2_	01–089–8939	trigonal	089281
	TiO_2_	01–088–1175	tetragonal	085495
	FeO	01–079–1973	cubic	067203
FeHJ-w	Fe_2_O_3_	01–089–0597	trigonal	082135
	Mn_2_(SiO_4_)	01–089–7714	orthorhombic	088026
	SiO_2_	01–089–8951	hexagonal	089293
	SiO_2_	01–083–0539	trigonal	079634
FeHJ	Fe_2_O_3_	01–072–0469	trigonal	015840
	Ca_2_Fe_2_O_5_	01–074–1860	orthorhombic	027808
	SiO_2_	01–089–8936	trigonal	089278
	SiO_2_	01–079–1915	trigonal	067126
FeHJ-2	Fe_2_O_3_	01–072–0469	trigonal	015840
	SiO_2_	01–089–8936	trigonal	089278
FeLJ	Fe_2_O_3_	01–073–0603	trigonal	022505
	Fe_3_O_4_	01–076–0958	orthorhombic	035003
	SiO_2_	01–085–0797	trigonal	027833
FeHP	Fe_2_O_3_	01–072–0469	trigonal	015840
	Fe_3_O_4_	01–089–0950	cubic	085806
	SiO_2_	01–085–0457	trigonal	016331
	SiO_2_	01–079–1915	trigonal	067126
HeHV	Fe_2_O_3_	01–089–0598	trigonal	082135
	Fe_3_O_4_	01–089–6466	orthorhombic	087697
	FeO	01–079–2179	cubic	067420
FeHL	Fe_2_O_3_	01–072–0469	trigonal	015840
FeTiHL	FeTiO_3_	01–071–1140	rhombohedral	009805
	MnO_2_	01–081–2261	tetragonal	073716
FeTiHM	FeTiO_3_	01–071–1140	rhombohedral	009805
	TiO_2_	01–075–1748	tetragonal	031321
	TiO	01–072–0020	monoclinic	015327

The dicalcium ferrite (Ca_2_Fe_2_O_5_), a bownmillerite structure, was also identified as an active phase in the FeHJ sample. According to the literature, Ca_2_Fe_2_O_5_ has the potential to be used as an oxygen carrier because it can be thermodynamically reduced to CaO and Fe^0^ in a single step, even in H_2_O and CO_2_ atmospheres, with complete regeneration of the crystal structure. In addition, bownmillerite is able to react selectively with methane (CH_4_) and increase H_2_ yield and promote stability over redox reactions.^40–42^

The pseudobrookite phase (Fe_2_TiO_5_), identified in FeLC-2 ore, is the oxidized form of ilmenite (FeTiO_3_) and acts as an active phase, since this solid has the ability to transfer oxygen through the reduction stages: Fe_2_TiO_5_ → Fe_2_TiO_4_ → FeTiO_3_. Under oxidizing conditions in CL processes, ilmenite assumes its highest oxidation state (Fe_2_TiO_5_) and is then reduced to FeTiO_3_ while delivering oxygen during the reaction with the fuel. The redox pair Fe_2_TiO_5_/FeTiO_3_ achieves a higher oxygen transport capacity (*R*_OC,Fe_2_TiO_5__ = 5.0%) compared to pure iron oxide (Fe_2_O_3_/Fe_3_O_4_ with *R*_OC,Fe_2_O_3__ = 3.4%).^26^

The ilmenite ores, FeTiHL and FeTiHM, have FeTiO_3_ oxide as the main crystalline phase. This phase was evaluated in CL processes for hydrogen production, demonstrating good reactivity, recyclability of the chemical structure over redox cycles, and high syngas conversion.^43^ In addition, the FeTiHL sample presented a small amount of manganese oxide (MnO_2_), which, under thermodynamically favorable reducing conditions, can act as an active phase, promoting a synergistic effect on the properties of this material. The FeTiHM sample, on the other hand, contains crystalline phases of titanium oxides (TiO_2_ and TiO), which do not contribute to the formation of ilmenite but can act as additives, improving the physicochemical characteristics of the material.

The morphological and surface chemical characteristics of the selected high-performance oxygen carriers were analyzed by SEM-EDS, as shown in Figure 5. The complete set of SEM-EDS images for all 13 iron ore samples is provided in the Supporting Information (Figure S1). In general, the particles exhibited slightly rough textures and irregular, pointed shapes, typical characteristics of iron ores.^44^

**Figure 5:**
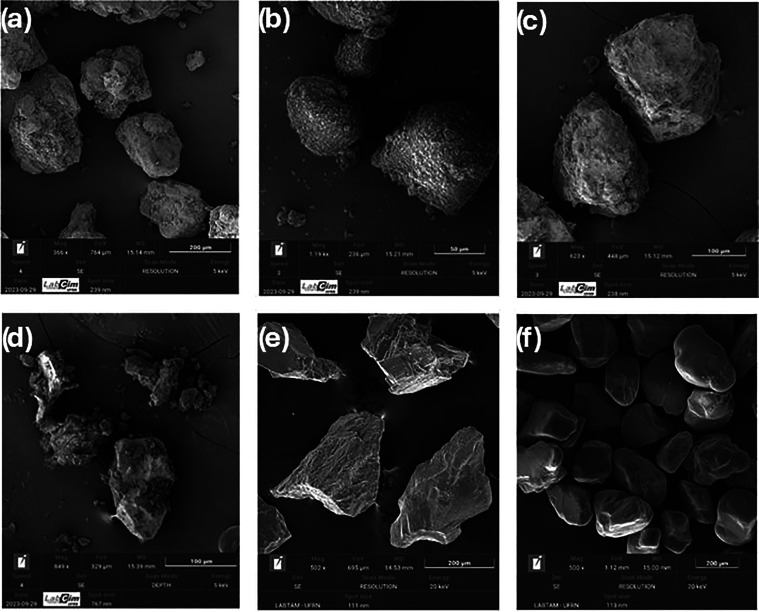
Imaging of the particles of the samples (a) FeHC, (b) FeLC, (c) FeHJ, (d) FeHP, (e) FeTiHL, and (f) FeTiHM by scanning electron microscopy (SEM).

The superficial mapping of the chemical composition performed by EDX revealed compositional diversity among the samples, corroborating the data obtained by XRF and XRD analyses. The FeHP sample (Figure 5d) showed the highest iron content among the hematite-based carriers, which is consistent with its superior oxygen transport capacity. The FeLC sample (Figure 5b) presented high percentages of silicon, while FeHC (Figure 5a) and FeHP (Figure 5d) revealed manganese contents (%Mn < 6.5%), which are in accordance with the XRF and XRD results. Additionally, the presence of calcium was identified in the chemical mapping of the FeHJ sample (Figure 5c). The complete chemical composition mapping for all samples, including FeHV, FeHL, FeLJ, FeHJ-w, FeHJ-2, FeMC, and FeLC-2, is presented in Figure S1 of the Supporting Information.

Figure 5e,f corresponds to the ilmenite ores (FeTiHL and FeTiHM, respectively), which exhibited distinct morphological characteristics. Figure 5e shows the FeTiHL particles, which were previously subjected to a calcination step to remove the organic binder introduced during the granulation stage, while providing mechanical strength to the granulated material. These particles exhibit agglomeration of smaller particles and rough surfaces. The surface chemical composition determined by EDX revealed contents of 55.88% Fe, 12.44% Ti, and 30.14% O, suggesting that calcination may have led to the migration of iron to the particle’s surface, forming an outer layer rich in iron.^45^ In contrast, Figure 5f corresponds to the micrograph of the FeTiHM in natural material, which shows particles with dense morphologies, rounded shapes, absence of visible pores or grains, and no signs of agglomeration. The surface chemical composition indicated contents of 24.0% Fe, 30.0% Ti, and 40.7% O, in addition to the presence of small amounts of impurities such as Zr, Al, Si, and Mn. The contrasting morphologies between FeTiHL and FeTiHM reflect their different processing histories and may contribute to their distinct reactivity profiles during redox cycles. The results of surface chemical composition obtained by EDX for ilmenites corroborate the data acquired by XRF and XRD, confirming the predominance of iron and titanium oxides, in addition to small levels of impurities.

TPR analysis was performed in order to characterize the reduction profiles. The results, grouped based on similar reduction profiles, are presented in Figure 6. Under similar reactional conditions, the literature reports that hematite exhibits three distinct reduction peaks: the first, at approximately 495 °C, is attributed to the reduction of Fe_2_O_3_ (hematite) to Fe_3_O_4_ (magnetite); the second, around 660 °C, corresponds to the reduction of Fe_3_O_4_ to FeO (wustite); and the third peak is related to the reduction of FeO to Fe^0^.^46,47^ It is important to note that the stages of hematite reduction are complex and can occur in a single way or in multiple stages, depending on the thermodynamic balance of iron oxides.^47,48^ Factors such as particle size, chemical composition, and crystallinity affect the reduction behavior, as well as the temperature ranges associated with each event.^6^ These variables may have caused variations in the observed reduction profiles in comparison with the patterns described in the literature.

**Figure 6:**
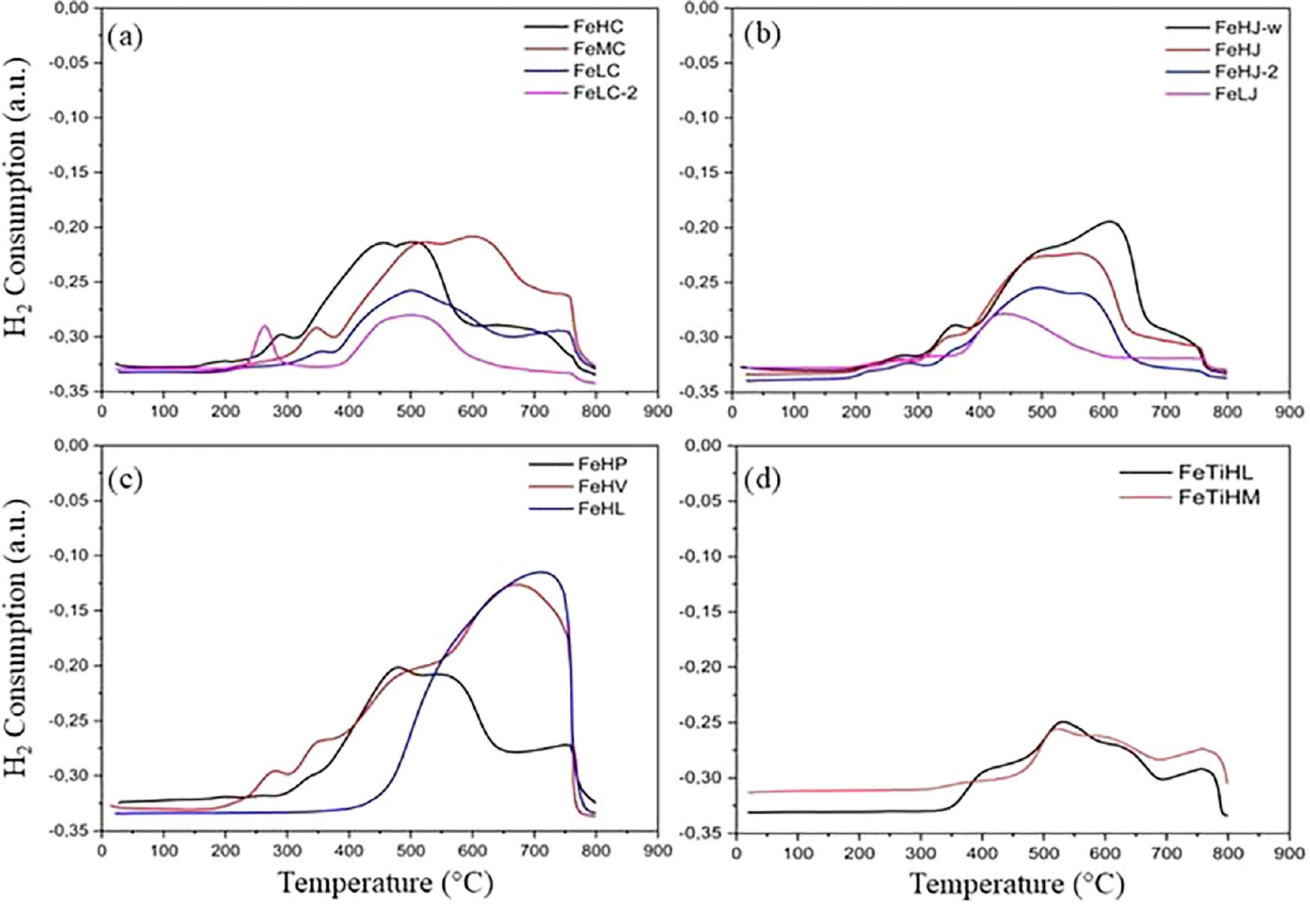
Temperature-programmed reduction (TPR) profiles: (a) Cruzeta ores, (b) Jucurutu ores, (c) other iron ores, and (d) ilmenite ores.

Figure 6 shows that the TPR patterns of FeHC, FeMC, FeHJ, FeHJ-2, FeHJ-w, and FeHP are very similar to three overlapping reduction bands corresponding to the hematite-reduction steps described by reactions 1–3.^6,46,47^ On the other hand, the ores FeLC and FeHP, containing iron oxides in different oxidation states (Fe^3+^, Fe^8/3+^, Fe^2+^), also showed three overlapping reduction bands, in accordance with previous reports. However, an increased intensity was observed in the magnetite reduction region (Fe_3_O_4_ → FeO → Fe). This behavior can be attributed to the additional contribution of the Fe_3_O_4_ phase formed from hematite, added to the magnetite originally present in these ores, as corroborated by the XRD results presented in Table 4.

R1 $${\mathrm{Fe}}_{3}O_{4}\to \mathrm{FeO}$$

R2 $${\mathrm{Fe}}_{3}O_{4}\to \mathrm{FeO}$$

R3 FeO→Fe

FeLC-2 ore, containing iron oxides (Fe^3+^ and Fe^2+^) and pseudobrookite (Fe_2_TiO_5_), showed a reduction profile with two well-defined events. The first event refers to Fe_2_O_3_ to Fe_3_O_4_ partial reduction, while the second, occurring between 390 and 700 °C, is attributed to the reduction of Fe_2_TiO_5_ to Fe^0^ (Fe_2_TiO_5_/FeTiO_3_/Fe_2_O_3_/Fe_3_O_4_/FeO/Fe), as well as the reduction in a single step of Fe_3_O_4_ to Fe^0^ (Fe_3_O_4_/FeO/Fe). Similarly, the FeLJ ore, which has iron oxides in its crystalline structure in the oxidation states Fe^3+^ and Fe^8/3+^, and FeHL, containing only hematite as the crystalline phase, exhibited single-step reduction profiles, corresponding to the direct reduction of Fe_2_O_3_ to Fe^0^. This behavior is consistent with results reported by Nascimento et al.^48^ Practically, all TPR profiles showed a small shoulder reduction at low temperatures, which is attributed to the reduction of superficial hematite to magnetite.^49^ This transformation occurs more readily due to the facilitated contact between hematite and H_2_.

Figure 6d shows the reduction profiles of ores that have ilmenite (FeTiO_3_) as the predominant phase. Considering that the FeTiHL sample had previously undergone a calcination stage (to remove the organic binder and increase its crushing strength), FeTiHM was also submitted to calcination under the same experimental conditions to enable a more accurate comparison between these materials. As evidenced by the XRD results of the natural ilmenite ores (Table 4), the main crystalline phase identified is FeTiO_3_. During the calcination process or under CL conditions, this phase is oxidized, as described in Reaction 4, to form pseudobrookite (Fe_2_TiO_5_), which is the desired active phase for redox cycle applications.

R4 $${\mathrm{FeTiO}}_{3}\left(\mathrm{FeO}+{\mathrm{TiO}}_{2}\right)\to 4\mathrm{FeO}+O_{2}+2{\mathrm{TiO}}_{2}\to 2{\mathrm{Fe}}_{2}O_{3}+2{\mathrm{TiO}}_{2}\to 2{\mathrm{Fe}}_{2}{\mathrm{TiO}}_{5}$$

Considering that TiO_2_ present in iron titanate is inert under TPR conditions, the reduction profiles of these mixed oxides are expected to originate TiO_2_ only from the ilmenite phase. Figure 6d reveals that FeTiHL ore has four reduction bands, while FeTiHM exhibits three reduction bands. The three overlapping events observed after 450 °C are attributed to the sequential reductions of Fe_2_O_3_ within the mixed oxides, as described in reaction 5. This process leads to the metallic iron and TiO_2_ formation, consequently promoting Fe_2_TiO_5_ segregation.^25,50,51^

R5 $${\mathrm{Fe}}_{2}{\mathrm{TiO}}_{5}\to {\mathrm{FeTiO}}_{3}\to {\mathrm{Fe}}_{2}O_{3}\to {\mathrm{Fe}}_{3}O_{4}\to \mathrm{FeO}\to {\mathrm{Fe}}^{0}$$

The FeTiHL sample has a reduction band around 400 °C, which can be attributed to the Fe^3+^/Fe^+8/3^ reduction of superficial Fe_2_TiO_5_/FeTiO_3_, which is easier to reduce. It should be noted that the incorporation of Ti into the Fe_2_O_3_ system increases the complexity of the reduction profile, with reduction events shifting to higher temperatures, as shown in Figure 6d.^52,53^ Table 5 presents temperature ranges associated with each reduction event as well as the total H_2_ consumption during the entire process.

**Table 5:** Temperature Ranges for Each Reduction Event for the Iron Ores Obtained from the TPR Results.

material/event	temperature			H_2_ consumption (g·cm^–3^)
	Fe_2_O_3_ → Fe_3_O_4_	Fe_3_O_4_ → 3FeO	FeO → Fe^0^	
FeHC	290–470 °C	470–617 °C	>620 °C	219.09
FeMC	300–547 °C	550–714 °C	>714 °C	319.05
FeLC	305–547 °C	547–660 °C	>660 °C	167.56
FeHJ-w	250–542 °C	532–680 °C	>680 °C	236.78
FeHJ	240–533 °C	533–709 °C	>709 °C	207.42
FeJl-2	250–532 °C	532–696 °C	>696 °C	144.86
FeHP	292–516 °C	516–662 °C	>662 °C	261.21
FeHV	222–370 (°C)	370–555 (°C)	>555(°C)	471.53
	Fe_2_O_3_ → Fe_3_O_4_	Fe_2_O_3_ → Fe^0^		
FeLC-2	230–310 °C	390–700 °C		77.74
	Fe_2_O_3_ → Fe^0^			
FeLJ	345–630 °C			80.32
FeHL	391–789 °C			399.57
	Fe_2_TiO_5_ → TiO_2_ + Fe^0^			
FeTiHL	350–790 °C			118
FeTiHM	470–800 °C			114

Considering that the main reaction in TPR experiments consists of Reaction 6 (M = metal atom) and that hydrogen consumption is directly related to the amount of oxygen present in the materials, it can be inferred that this consumption is proportional to the oxygen transfer capacity in chemical looping processes.

R6 $$M_{x}O_{y}+H_{2}\to xM+{yH}_{2}O$$

### 3.2 Oxygen Carrier Preselection

FeHC, FeLC, FeLC-2, FeHJ, FeHJ-2, FeHP, FeHL, FeTiHL, and FeTiHM ores were selected for reactivity evaluation in the thermobalance due to their high crushing strength (CS > 1 N) and their crystalline structures, which can act as active phases in the CL process. All selected ores predominantly contain hematite or pseudobrookite (Fe_2_TiO_5_) as active phases, with variations in the percentage of the active phase and the presence of secondary active phases. Only FeLC-2 and FeHJ ores showed secondary active phases.

FeLC-2 contains the crystalline phase pseudobrookite (Fe_2_TiO_5_), the oxidized form of ilmenite (FeTiO_3_), which is of significant interest in CL processes due to its high oxygen transport capacity. FeHJ contains the crystalline phase Ca_2_Fe_2_O_5_, which has potential as an oxygen carrier as it can be thermodynamically reduced from Ca_2_Fe_2_O_5_ to CaO and Fe^0^ in a single step, besides being able to be completed regenerated during the oxidation step, promoting cyclic stability over redox reactions.^6^

FeHP was selected due to its high H_2_ consumption during TPR analysis, aiming to evaluate the influence of the mixture of iron oxides in different oxidation states (Fe^3+^, Fe^8/3+^, and Fe^2+^). FeHL was selected due to its high purity and crystallinity.

Therefore, the selected samples will be evaluated on the thermobalance to determine their oxygen transport capacity and reactivity with methane, hydrogen, and oxygen gases.

### 3.3 Oxygen Carrier Reactivity Experiments

The oxygen transport capacity parameter (*R*_oc_) depends on the type of oxide and the degree of reduction that the oxygen carrier (OC) material can achieve. The degree of oxidation is determined by the thermodynamic conditions of the reaction medium, controlled by the addition of water vapor to the reducing gas mixture. For example, for iron-based OCs, a gas composition containing 5% H_2_ and 40% H_2_O thermodynamically limits the reduction of the active phases to the Fe_2_O_3_/Fe_3_O_4_ pair. On the other hand, in a composition with 15% H_2_ and 20% H_2_O, the reduction can proceed to FeO (Fe_2_O_3_/Fe_3_O_4_/FeO).^6,54^ Under conditions of absence of water vapor (15% H_2_), the reduction of iron oxide is complete (Fe_2_O_3_/Fe_3_O_4_/FeO/Fe^0^).^6^ In the case of ilmenite-based oxides (Fe_2_TiO_5_), the gas compositions mentioned above are not sufficient to limit the reduction. Regardless of the presence or concentration of water vapor, ilmenite oxides are completely reduced to FeTiO_3_.^24^

During the redox cycles carried out in the thermobalance, the main active phase of the iron-based OCs was Fe_2_O_3_ (Table 4). The mass variations recorded in the thermograms correspond to the conversions of the OCs via the following process: Fe_2_O_3_ → Fe_3_O_4_ → FeO → Fe, as described in Reactions 7–9 for the reduction step and in Reactions 11–13 for the oxidation step. Additionally, the conversions of the ilmenite-based OCs resulted in mass transfers related to Reaction 10 in the reduction step and Reaction 14 in the oxidation step.

R7 $$12{\mathrm{Fe}}_{2}O_{3}+{\mathrm{CH}}_{4}\to 8{\mathrm{Fe}}_{3}O_{4}+{\mathrm{CO}}_{2}+2H_{2}O$$

R8 $$4{\mathrm{Fe}}_{3}O_{4}+{\mathrm{CH}}_{4}\to 12\mathrm{FeO}+{\mathrm{CO}}_{2}+2H_{2}O$$

R9 $$4{\mathrm{Fe}}_{2}O_{3}+{\mathrm{CH}}_{4}\to 8\mathrm{FeO}+{\mathrm{CO}}_{2}+2H_{2}O$$

R10 $$4{\mathrm{Fe}}_{2}{\mathrm{TiO}}_{5}+4{\mathrm{TiO}}_{2}+{\mathrm{CH}}_{4}\to 8{\mathrm{FeTiO}}_{3}+{\mathrm{CO}}_{2}+2H_{2}O$$

R11 $$\mathrm{Fe}+1/2O_{2}\to \mathrm{FeO}$$

R12 $$3\mathrm{FeO}+1/2O_{2}\to {\mathrm{Fe}}_{3}O_{4}$$

R13 $$4{\mathrm{Fe}}_{3}O_{4}+O_{2}\to 6{\mathrm{Fe}}_{2}O_{3}$$

R14 $$4{\mathrm{FeTiO}}_{3}+O_{2}\to 2{\mathrm{Fe}}_{2}{\mathrm{TiO}}_{5}+2{\mathrm{TiO}}_{2}$$

Figure 7 presents the results of *R*_oc_ evolution during the redox cycles of the selected ores, represented by the bars. It is observed that OCs FeLC-2, FeHJ, and FeHL presented a constant *R*_oc_ as a function of the cycles. In contrast, the OCs FeHJ-2, FeHC, FeLC, and FeHP showed a progressive decrease in oxygen transport capacity (*R*_oc_) over the cycles. This decrease can be attributed to an initial stabilization step of the OCs FeHJ-2, FeHC, FeLC, and FeHP. Although these materials fully regenerate after the oxidation step, in experiments carried out in an atmosphere containing 5% H_2_ + 40% H_2_O, the percentage of active phases for subsequent cycles decreases, resulting in a gradual reduction of *R*_oc_. In this context, the *R*_oc_ of the third cycle was fixed for the calculation of the conversion of the OCs during reduction (*X*_red_) and oxidation (*X*_oxi_) with a reactive atmosphere of 15% CH_4_ + 20% H_2_O. Thus, the evolution of solids conversion (X_red_ and X_oxi_) can also be seen in Figure 7. The data indicates that the samples FeLC-2, FeHJ-2, FeHC, FeHL, and FeHP showed a constant increase in solids conversion, with the exception of FeHP, which showed a reduction in its conversion during the third redox cycle.

**Figure 7:**
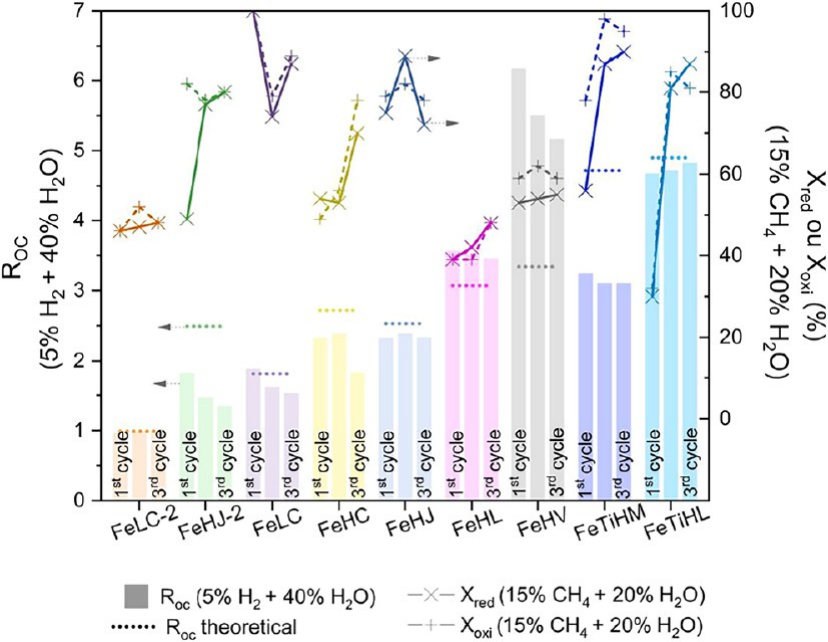
Results of *R*_OC_ evolution and conversion during reduction and oxidation over the course of the 3 redox cycles carried out in a thermobalance with a feed of 15% CH_4_ + 20% H_2_O.

Figure 7 also shows that among the ilmenite samples, FeTiHL presented a constant *R*_OC_ in the second and third cycles, while FeTiHM in natura showed a slight increase in oxygen transport capacity over the cycles. This behavior suggests that initially, the FeTiHM sample is oxidized under the reaction conditions, reaching the higher oxidation state (Fe_2_TiO_5_), which has a higher theoretical *R*_OC_ and constitutes the phase of interest for the chemical looping process. Additionally, an increase in solid conversion was observed over the redox cycles in the reduction step (*X*_red_), and a stabilization from the second to the third cycle in the oxidation step (*X*_oxi_).

The regeneration performance of the oxygen carriers was assessed over three redox cycles and is illustrated in Figure 7, which shows the *X*_oxi_ values for each cycle. In addition, to analyze in more detail the solid conversion as a function of time and the reactivity of the materials in the third cycle, in both the reduction and oxidation steps, the results are presented in Figure 8a–d. The trends observed indicate that all nine samples underwent complete regeneration throughout the cycles. The main results obtained for the selected iron-based oxygen carriers are summarized in Table 6, which also provides the values of the Rate Index during the oxidation step (ranging from 2.11 to 12.28 among the samples). These results suggest that, while all oxygen carriers were successfully reoxidized, some exhibited faster oxidation kinetics (FeHP > FeHL > FeTiHL > FeHJ), whereas others followed slower kinetic profiles (FeHC > FeHJ-2 > FeLC > FeTiHM > FeLC-2).

**Figure 8:**
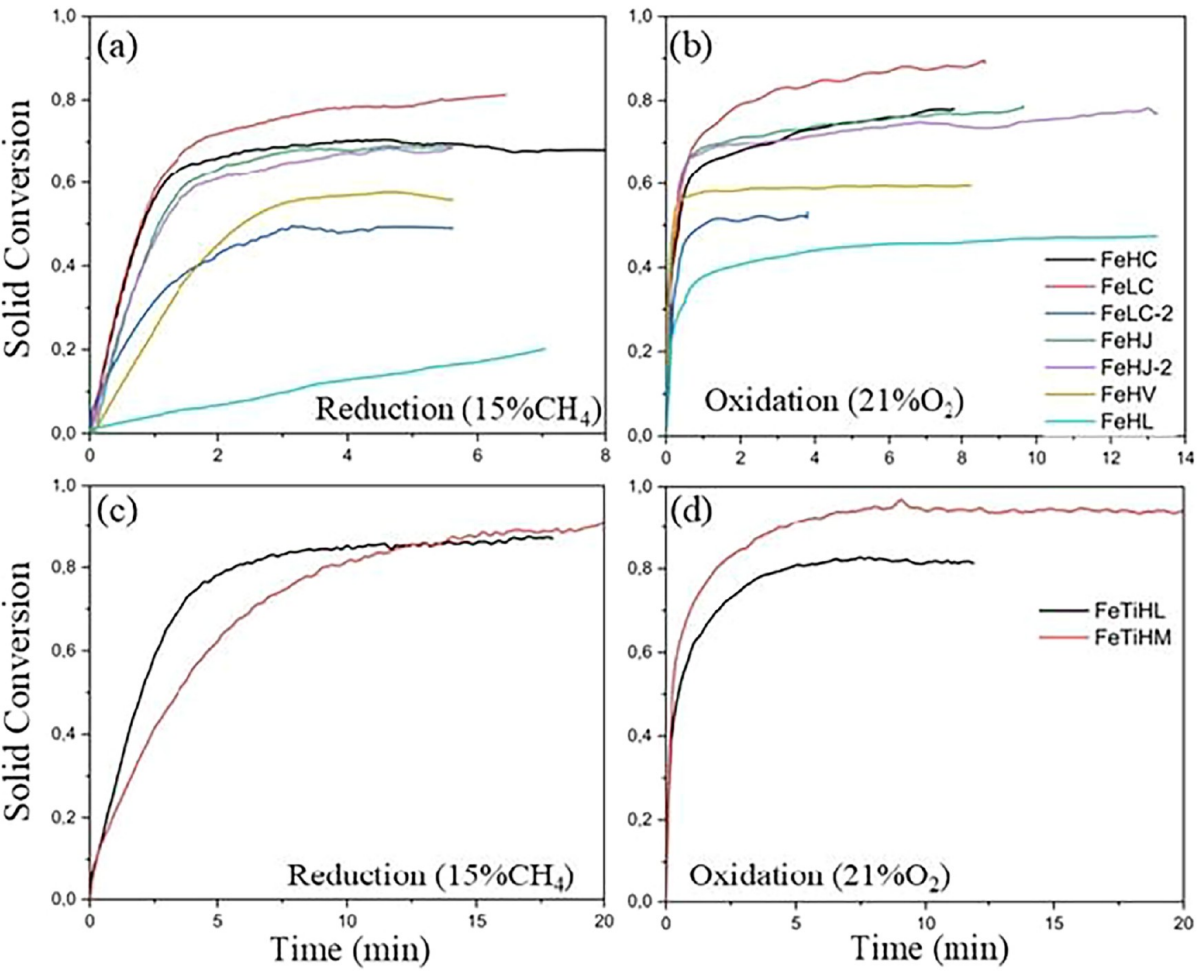
Reactivity results with CH_4_ of the iron-based oxygen carriers: conversion curves (a) during reduction, (b) during oxidation, (c) ilmenite during reduction, and (d) ilmenite during oxidation.

**Table 6:** Reactivity Results with CH_4_ of the Selected Iron Ores.

				reactivity (15% CH_4_ + 20% H_2_O)				reactivity (15% H_2_ + 20% H_2_O)			
		determination of parameters (5% H_2_ + 40% H_2_O)		reduction		oxidation		reduction		oxidation	
material	*R*_OC_^a^ theoretical (%)	*R*_OC_ (%)	RI_oxi_^b^ (%/min)	*X*_red_ (%)	RI_red_ (%/min)	*X*_oxi_ (%)	RI_oxi_ (%/min)	*X*_red_ (%)	RI_red_ (%/min)	*X*_oxi_ (%)	RI_oxi_ (%/min)
FeHC	2.72	1.83	4.39	70	1.08	78	2.47	187	4.40	189	8.67
FeLC	1.82	1.52	3.06	87	0.91	89	1.86	214	4.44	208	5.57
FeLC-2	1.21	0.99	2.11	48	0.31	48	1.33				
FeHJ	2.53	2.33	6.90	72	1.25	78	5.26	156	6.58	157	7.90
FeHJ-2	2.49	1.34	3.34	80	0.62	80	1.88				
FeHP	3.34	5.16	12.28	55	1.34	59	11.98	1.38	10.78	1.38	12.51
FeHL	3.07	3.45	8.54	48	0.12	48	2.97				
FeTiHM	4.72	3.10	2.99	90	0.70	95	3.72	112	3.39	116	6.53
FeTiHL	4.90	4.82	7.12	87	1.16	81	7.83	96	7.98	98	9.20

Among the samples from the Cruzeta region, FeLC stands out, which presented a maximum conversion of approximately 80%. In addition, the highest oxygen transfer rates were observed for the FeLC and FeHC samples, evidenced by the steeper slope of the conversion curve as a function of time (Figure 8a). These results suggest that the active phase Fe_2_TiO_5_ present in the FeLC-2 sample did not show significant activation, not contributing to the reactivity of this oxygen carrier (OC). Thus, it became evident that the FeLC sample, characterized by its low iron content and chemical composition with impurities such as silica and alumina, stood out among the other samples from the Cruzeta region. Its high solids conversion and high reaction rate indicate potential for industrial applications.

The FeHJ and FeHJ-2 samples (Jucurutu region) presented similar conversions (Table 6). However, FeHJ showed a higher yield of the reducible active phases (experimental *R*_OC_ closer to theoretical *R*_OC_) and high rate index values in the reduction and oxidation steps, indicating the synergistic effect that the Ca_2_Fe_2_O_5_ phase provided. Calcium ferrite Ca_2_Fe_2_O_5_ is chemically stable and presents good reducibility, high oxidation activity, and high cyclical stability. Additionally, calcium can positively influence the reduction of Fe^3+^ to Fe^0^, promoting simple and efficient reactions composed of a single step.^55^

Despite the high crystallinity and oxygen transport capacity, FeHL showed low solid conversion and slow reaction rates during the reduction step with CH_4_ (RI_CH_4__ = 0.12). On the other hand, the FeHP sample, composed of mixtures of crystalline phases of iron oxides in different oxidation states, exhibited the highest experimental oxygen transport capacity and the highest reaction rate in the oxidation step among all of the evaluated samples. However, the low solids conversion of the FeHP sample with CH_4_ gas suggests that it is still in the process of activation and that its cyclical stability has not been completely achieved. Previous studies report that iron ores can undergo an activation process over redox cycles until reaching operational stability.^56^ In this sense, it is expected that the solid conversion will increase with the performance of successive redox cycles, making it necessary to further investigate the behavior of this sample.

For the ilmenite samples, the chemical stress associated with the redox reactions influences the reaction rate, promoting an activation until a maximum oxygen transfer rate is reached, which remains constant over the cycles.^26^ In this context, the oxygen carrier FeTiHL stands out, which has already undergone the activation process, presenting a higher *R*_OC_, greater utilization of the reducible active phases, and high rate index (RI) values in the reduction and oxidation steps, when compared to the in natura ilmenite FeTiHM, which is still in the activation process (characterized by the constant increase in *R*_OC_). Additionally, the results suggest that manganese oxide present in the crystalline structure of FeTiHL may contribute to the increase in oxygen transport capacity, exerting a synergistic effect with the Fe_2_TiO_5_ phase. Based on the presented results, it is possible to affirm that all of the evaluated OCs fully regenerate during the oxidation step. Furthermore, the oxidation kinetics were faster than the reduction kinetics, as evidenced by the higher RI values in the oxidation step (RI_oxi‑CH_4__).

Therefore, considering the highest solids conversions associated with the most reactive carriers with methane (RI_CH_4__ > 1), the following carriers stand out, in decreasing order of reactivity: FeHP, FeHJ, FeTiHL, FeHC, FeLC, and FeTiHM. These materials are promising for application in CL processes for combustion of gaseous fuels (*e.g*., natural gas) and for solid fuels^57^ and were selected to proceed with reactivity tests with 15% H_2_ + 20% H_2_O, as shown in the flowchart presented in Figure 2.

Figure 9 presents the solid conversion of the third redox cycle with hydrogen gas (15% H_2_ + 20% H_2_O). It was observed that the OCs FeHP, FeHJ, FeHC, and FeLC obtained conversions above 100% (*X*_red_ > 1), indicating the progression of the reduction of the Fe_2_O_3_–Fe_3_O_4_–FeO phases.^58^ During the three redox cycles with H_2_, the total conversion of the OCs was achieved due to the fast reduction rate of Fe_2_O_3_ to Fe_3_O_4_, followed by the continuation of the reduction reaction with the conversion of Fe_3_O_4_ to FeO, although with slower reaction rates.^6,59^ This difference in reaction rates between the two stages can be attributed to different control mechanisms. In the first stage, the reduction is controlled by the diffusion of the reactant gas through the outer layer of the particle surface, while in the second stage, the control is predominantly exerted by heterogeneous chemical reactions.^60^

**Figure 9:**
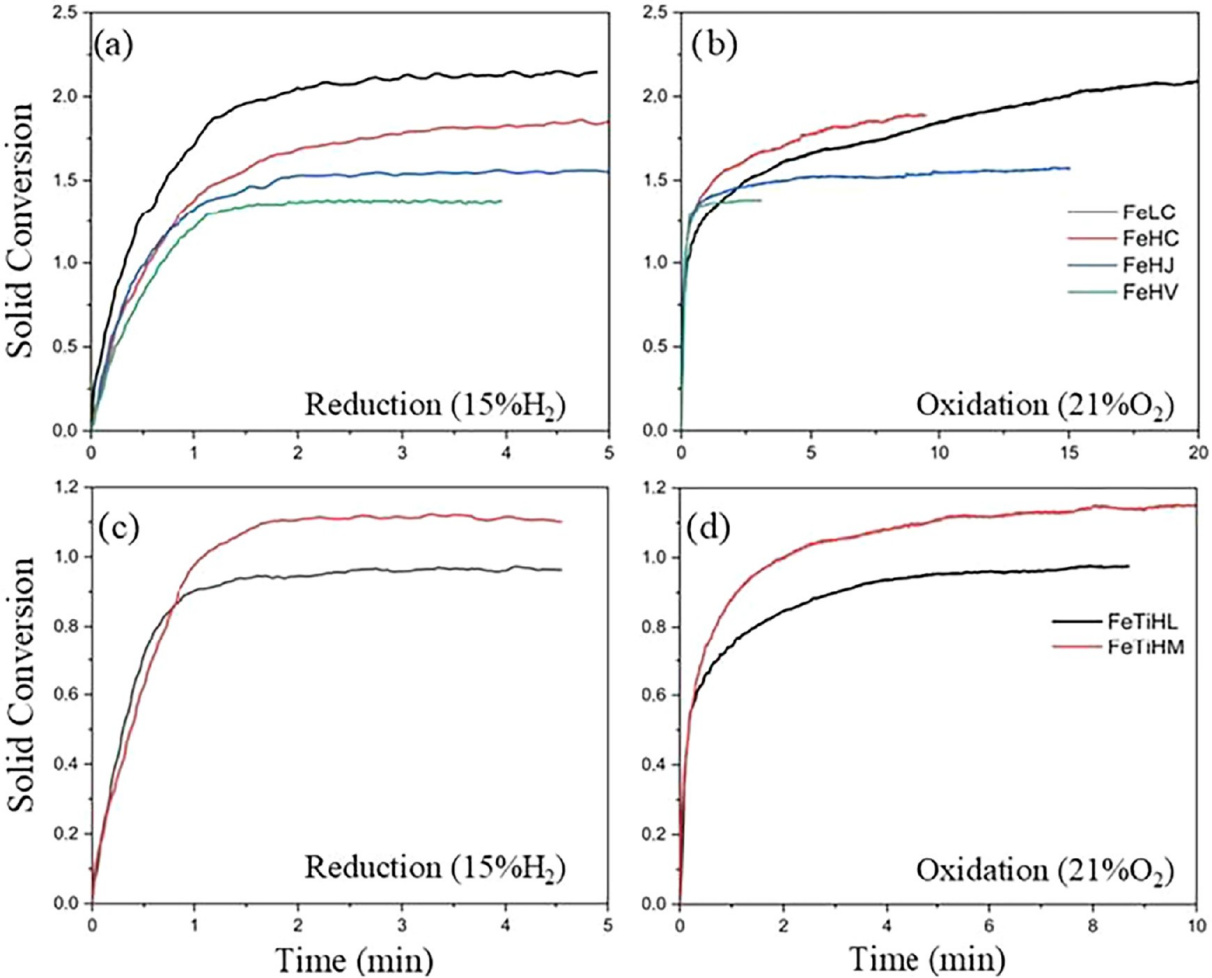
Reactivity results with H_2_ of the iron-based oxygen carriers: conversion curves (a) during reduction, (b) during oxidation, (c) ilmenite during reduction, and (d) ilmenite during oxidation.

The ilmenite samples FeTiHL and FeTiHM showed stability in solid conversion over the redox cycles with H_2_ gas. However, the solid conversion of the FeTiHL sample reached approximately 100% in the reduction and oxidation steps, as seen in Table 6. This conversion indicates that the reduction proceeded through Fe_2_TiO_5_/FeTiO_3_ and Mn_2_O_3_/MnO. Considering that calcination processes can promote the migration of iron to the particle surface, forming an iron-rich outer layer whose thickness increases with the number of redox cycles performed, a limited reduction can minimize the segregation of iron ions.^26^ On the other hand, the in natura FeTiHM sample showed higher conversions, but with slower reaction rates, suggesting that the reduction proceeded through a more complete path: Fe_2_TiO_5_ → FeTiO_3_ → Fe + TiO_2_. In this case, the first reduction stage (Fe_2_TiO_5_ → FeTiO_3_) occurs relatively quickly, while the subsequent steps proceed at slower reaction rates.

During the oxidation step, for all evaluated oxygen carriers (OCs) (Figures 8 and 9), it was observed that the conversions (*X*_oxi_) correspond to those obtained in the reduction step within a short time interval. All of the mass lost in the reduction step was recovered in the oxidation step, evidencing complete regeneration of the OCs. The most interesting aspect of this result is that, contrary to what is reported in the literature, even with the formation of FeO, complete regeneration and stability among the three redox cycles were achieved in a short period. Furthermore, as observed in Table 6, the reaction rates with hydrogen gas were significantly higher compared to methane gas, due to the high diffusion rate of hydrogen, which allows its more efficient penetration into the crystalline structure of the oxygen carriers.^61^

To verify the contribution of the active phases in the OCs FeHJ and FeTiHL (Table 4) to oxygen transfer, the *R*_OC_ of each reducible phase was determined experimentally, through the system of equations obtained by equating the parameters found under the conditions of 5% H_2_ + 40% H_2_O and 15% H_2_ + 85% N_2_, according to eqs 19 and 20. Thus, for the FeHJ sample, the Fe_2_O_3_ phase presents an *R*_OC_ equivalent to 1.41%, and the manganese oxide phase revealed an *R*_OC_ equal to 0.91%, totaling *R*_OC_ = 2.33% (Table 6). As for the ilmenite sample FeTiHL, the FeTiO_3_ phase contributes with 2.74% of oxygen transport capacity, and the manganese oxide contributes with *R*_OC_ = 2.08%, totaling *R*_OC_ = 4.82% (Table 6). Corroborating the data presented above, the contribution of two different phases leads to a synergistic effect, directly impacting the reactivity of the OCs. This can be better evaluated over multiple redox cycles.

For 5% H_2_ + 40% H_2_O:

5 $$\%f\mathrm{active phase}\;x\;Ro_{\left(5\%H_{2}\right)}={\%}_{{Fe/\mathrm{FeTiO}}_{3}}\;x\;Ro_{\left({Fe}_{2}O_{3}/{Fe}_{3}O_{4}-{Fe}_{2}{TiO}_{5}/{FeTiO}_{3}\right)}+{\%}_{Mn/{Ca}_{2}{Fe}_{2}O_{5}\;}\;x\;Ro_{\left({Mn}_{2}O_{3}/MnO-{Ca}_{2}{Fe}_{2}O_{5}/CaO+Fe\right)}$$

For 15% H_2_:

6 $$\%f\mathrm{active phase}\;x\;Ro_{\left(15\%H_{2}\right)}={\%}_{{\mathrm{Fe}/\mathrm{FeTiO}}_{3}}\;x\;Ro_{\left({\mathrm{Fe}}_{2}O_{3}/\mathrm{Fe}-{\mathrm{Fe}}_{2}{\mathrm{TiO}}_{5}/{\mathrm{FeTiO}}_{3}\right)}+{\%}_{\mathrm{Mn}/{\mathrm{Ca}}_{2}{\mathrm{Fe}}_{2}O_{5}}\;x\;Ro_{\left({\mathrm{Mn}}_{2}O_{3}/\mathrm{MnO}-{\mathrm{Ca}}_{2}{\mathrm{Fe}}_{2}O_{5}/\mathrm{CaO}+\mathrm{Fe}\right)}$$

The thermodynamic restriction of the reduction of hematite to magnetite (Fe_2_O_3_ → Fe_3_O_4_) is a relevant strategy in the chemical looping combustion (CLC) process, as it favors complete combustion, resulting in the obtaining of carbon dioxide (CO_2_) with high purity. In contrast, achieving the oxidation states wustite (FeO) and metallic iron (Fe^0^) is particularly advantageous in processes such as chemical looping reforming (CLR) and chemical looping gasification (CLG), where incomplete combustion promotes the increase in the concentration of synthesis gas (CO and H_2_).^1^ In continuous processes, complete or incomplete combustion can be controlled and adjusted through operational parameters, such as the air flow used to promote the reoxidation of the oxygen carrier (OC) in the air reactor (AR) or by the recirculation rate of the OC between the air (AR) and fuel (FR) reactors. However, the reoxidation of the wustite (FeO) and metallic iron (Fe^0^) phases is often associated with agglomeration problems, due to the volumetric expansion and coalescence of the materials, which can compromise operational stability.^6,46,62^

Based on the reactivity results obtained by thermogravimetry, the materials FeHP, FeHJ, FeTiHL, FeHC, FeLC, and FeTiHM stood out significantly in their interaction with methane, demonstrating their potential for application in chemical looping processes. These OCs also showed considerably higher reactivities than expected when tested with H_2_, suggesting their suitability for chemical looping-assisted reforming or gasification (CLG) processes. The high percentage of reactivity with H_2_ indicates that these materials were mainly reduced to FeO or metallic Fe, which, although undesirable for applications in fluidized bed reactors due to the possible agglomeration resulting from their oxidation, can be efficiently mitigated by operational adjustments, such as controlling the solid circulation rate and/or modulating the fuel feed.

### 3.4 Characterization of Materials after Reactive Processes

The selected materials were subjected to X-ray diffraction (XRD) analysis after temperature-programmed reduction and reactivity processes in the thermobalance. The objective was to understand the changes in the crystalline phases that occur in the oxygen carriers after reactive processes. The obtained diffractograms are presented in Figure 10, in which the phases corresponding to the inert materials (mainly SiO_2_) were excluded for better visualization.

**Figure 10:**
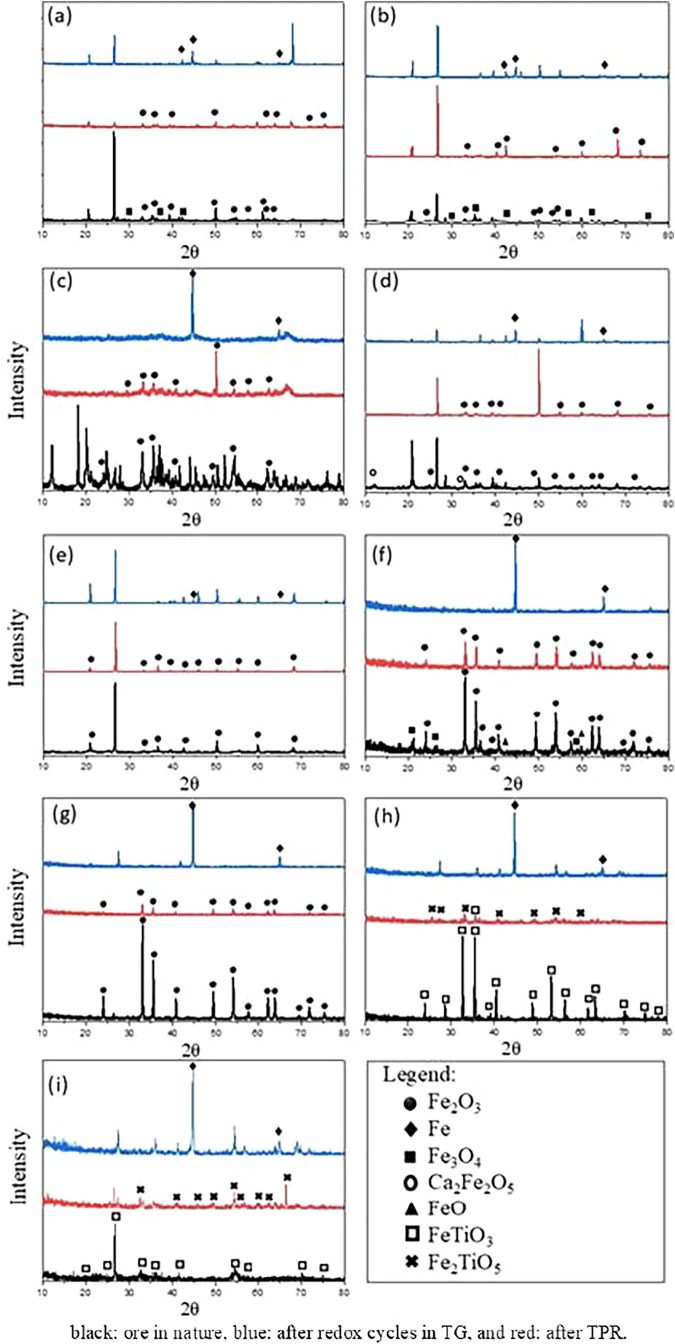
X-ray diffraction patterns of the selected iron ores: in natura (black), post-TG (red), and post-TPR (blue). (a) FeHC, (b) FeLC, (c) FeLC-2, (d) FeHJ, (e) FeHJ-2, (f) FeHP, (g) FeHL, (h) FeTiHL, and (i) FeTiHM.

After the completion of the reactivity cycles carried out in the thermobalance, the exclusive presence of the hematite phase (Fe_2_O_3_) was verified, indicating that the magnetite and wustite phases, observed in some in natura samples, were completely oxidized at the end of the experiment. This behavior was expected and demonstrates that all iron oxides present in the OCs were completely reoxidized to their highest oxidation state, with the Fe_2_O_3_ phase being the most thermodynamically stable under the operational conditions used.^63^ Thus, it is confirmed that the particles did not undergo significant changes in their phases and crystalline structure throughout the process.

Similarly, it was found that the conditions of the temperature-programmed reduction (TPR) test were sufficient to reduce the iron oxides of all of the ores to metallic iron. This behavior was also expected, since the oxygen carriers (OCs) were subjected to a reducing atmosphere of hydrogen, in the absence of water vapor, and at temperatures above 400 °C.^64^ Additionally, it was observed that the Ca_2_Fe_2_O_5_ phase, present in the in natura FeHJ sample, was not identified after the thermogravimetry (TG) and TPR tests. This result suggests that segregation of this mixed oxide into its constituent simple oxides occurred due to the experimental conditions.

The predominant active phase in the FeTiHL and FeTiHM samples was identified as ilmenite (FeTiO_3_), while the MnO_2_ phase was detected exclusively in the FeTiHL sample. However, after the third redox cycle performed in the thermogravimetric reactivity (TG) experiments with CH_4_ and H_2_ gas, under oxidizing conditions, it was found that part of the bivalent iron (FeTiO_3_) was transformed into trivalent iron (Fe_2_O_3_) and titanium oxide (TiO_2_). This transformation can influence the redox behavior of the samples, considering that the presence of bivalent iron (Fe^2+^) is associated with a higher oxygen transport capacity. Additionally, the conditions of the temperature-programmed reduction (TPR) test were sufficient to reduce ilmenite to metallic iron (Fe^0^) and titanium oxide (TiO_2_).

The results presented in Figure 10 are summarized in Table 7 with the iron phases identified in the XRD of the in natura, oxidized post-TG with CH_4_ gas, and reduced post-TPR experiments with H_2_ gas samples of the selected oxygen carriers.

**Table 7:** Crystalline Phases of the Iron-Based Oxygen Carriers Obtained from the X-ray Diffractograms of the In Natura Oxidized After-TG and Reduced After-TPR Particles.

material	*in natura*	after TG–CH_4_/H_2_	after TPR
FeHC	Fe_2_O_3_	Fe_2_O_3_	Fe
	Fe_3_O_4_		
FeLC	Fe_2_O_3_	Fe_2_O_3_	Fe
	Fe_3_O_4_		
FeLC-2	Fe_2_O_3_	Fe_2_O_3_	Fe
	FeO		
	Fe_2_TiO_5_		Fe_2_TiO_5_
FeHJ	Fe_2_O_3_	Fe_2_O_3_	Fe
	Ca_2_Fe_2_O_5_		
FeHJ-2	Fe_2_O_3_	Fe_2_O_3_	Fe
	Fe_2_O_3_		
	Fe_3_O_4_		
FeHP	Fe_2_O_3_	Fe_2_O_3_	Fe
	Fe_3_O_4_		
	FeO		
FeHL	Fe_2_O_3_	Fe_2_O_3_	Fe
FeTiHM	FeTiO_3_	Fe_2_O_3_	FeTiO_3_
	TiO_2_	Fe_2_TiO_5_	Fe
	TiO	TiO_2_	TiO_2_
FeTiHL	FeTiO_3_	Fe_2_O_3_	Fe
	MnO_2_	Fe_2_TiO_5_	TiO_2_
		TiO_2_	

The results presented in Figure 10 and summarized in Table 7 corroborate the observations discussed in the temperature-programmed reduction (TPR) and thermogravimetric reactivity (TG) analyses. For materials containing simple iron oxides, it was found that the most oxidized phase formed is predominantly hematite (Fe_2_O_3_). Depending on the composition of the reactive gas, these materials can be reduced to magnetite (Fe_3_O_4_), as observed in atmospheres containing 5% H_2_ + 40% H_2_O or methane. Under conditions of higher reducing gas concentrations, the reduction can proceed to wustite (FeO) or metallic iron (Fe).

On the other hand, ilmenites, under the evaluated operational conditions, form the most oxidized phase Fe_2_TiO_5_ (pseudobrookite). These materials can be reduced to FeTiO_3_ (ilmenite) or, under extreme conditions, undergo segregation, resulting in metallic iron and TiO_2_. These behaviors indicate that the composition and reaction conditions play crucial roles in the stability of the oxidizing and reducing phases.

### 3.5 Application Recommendations for Different Chemical Looping Processes

Based on the comprehensive characterization of the iron ore oxygen carriers, specific recommendations can be provided for their optimal application in different chemical looping processes. The selection criteria considered reactivity, oxygen transport capacity, cyclic stability, and operational requirements.

#### 3.5.1 Chemical Looping Combustion (CLC)

For CLC applications targeting energy generation, the FeHC, FeLC, and FeTiHL samples are recommended due to their high reactivity with methane and excellent oxygen transfer rates. FeLC demonstrated the highest solid conversion (∼80%) among hematite-based carriers and a high RI_red_ of 0.91%·min^–1^ for CH_4_, while FeTiHL showed superior cyclic stability after the activation process, with a high ROC (4.82%) and rapid oxidation (RI_oxi_ of 7.83%·min^–1^), suggesting favorable kinetics for the fast transfer of oxygen required for combustion. Operating conditions should include a temperature of 900 °C in the reactor, with fuel concentrations of 15% CH_4_ and residence times of 2–4 min.

#### 3.5.2 Chemical Looping Reforming (CLR)

For syngas production via CLR, FeHJ and FeHJ-2 samples are particularly suitable due to the synergistic effect of the Ca_2_Fe_2_O_5_ phase, which enhances cyclic stability and reduces coke formation. The recommended operating conditions include temperatures of 900–1000 °C, steam-to-carbon ratios of 1.5–3.0, and controlled water vapor addition to optimize H_2_/CO ratios between 1.5 and 2.5.

#### 3.5.3 Chemical Looping Water Splitting (CLWS)

For hydrogen production through CLWS, FeHP is the most promising candidate due to its highest experimental ROC (5.16 wt % for the 5% H_2_ + 40% H_2_O test) and exceptionally fast oxidation kinetics (RIoxi of 11.98%·min^–1^ with CH_4_ and 12.51%·min^–1^ with H_2_), which is critical for the rapid regeneration step involving steam.. However, this material requires an initial activation period of 3–5 cycles to achieve stable performance. Operating temperatures should range from 950 to 1100 °C during reduction and 800–900 °C during steam oxidation.

#### 3.5.4 Chemical Looping Gasification (CLG)

For biomass gasification applications (CLG), FeTiHM and FeHJ-2 are recommended. FeTiHM shows progressive activation during redox cycles, reaching a high X red of 90% and demonstrating RIred values of 3.39%·min^–1^ (with H_2_), indicating its potential for deeper reduction, necessary for syngas production. FeHJ-2 also stands out for its superior cyclic stability and high conversion (X red of 80%).

The selection matrix presented in Table 8 summarizes the optimal applications for each oxygen carrier based on their characterized properties.

**Table 8:** Selection Matrix for Oxygen Carrier Applications

oxygen carrier	primary process^a^	secondary process^a^	recommended temperature (°C)	*R*_oc_ (%)	special observations
FeHC	CLC	CLR	850–950	2.85	high reactivity with CH_4_ ^29^
FeLC	CLC	CLWS	800–900	3.21	excellent conversion (>80%)^64^
FeHJ	CLR	CLC	900–1000	2.95	synergistic effect with Ca_2_Fe_2_O_5_ ^65^
FeHJ-2	CLR	CLG	850–950	2.78	superior cyclic stability^24^
FeHP	CLWS	CLC	950–1100	4.90	higher ROC, requires activation^62^
FeHL	CLR	CLR	800–900	1.85	high purity, low CH_4_ reactivity^66^
FeTiHL	CLC (solids)	CLG	900–1050	3.85	preactivated, high stability^26^
FeTiHM	CLG	CLC	850–950	2.15	progressive activation^67^

a CLC: chemical looping combustion; CLR: chemical looping reforming; CLWS: chemical looping water splitting; CLG: chemical looping gasification.

## 4 Conclusion

The systematic evaluation of 13 Brazilian iron ores demonstrated their high viability as oxygen carriers (OCs) for chemical looping processes. The predominant active phases, hematite and ilmenite, exhibited efficiency in oxygen transport and high reactivity under the experimental conditions.

The materials FeHP, FeHJ, FeHC, FeLC, FeTiHL, and FeTiHM stood out, combining high oxygen transport capacity (ROC), reactivity with CH_4_ and H_2_, cyclical stability, and fracture resistance >2.2 N. The results suggest that adjustments in operational conditions, particularly the use of operating temperatures in the range of 800–1100 °C for the distinct CL processes (as detailed in the manuscript), can further optimize the performance of these materials, expanding their use in chemical looping combustion (CLC) and in chemical looping-assisted reforming and gasification (CLR and CLG). This study contributes to the advancement of chemical looping technologies, highlighting the potential of Brazilian ores as sustainable, economical, and high-efficiency solutions for the energy sector, promoting promising technological alternatives for CO_2_ capture and utilization and the reduction of environmental impact in the industrial and energy sectors.

## Supplementary Material



## Notes

Supporting discussion regarding the morphological and surface chemical characterization of all iron ore samples; comprehensive scanning electron microscopy (SEM) imaging and energy-dispersive X-ray spectroscopy (EDX) chemical mapping of all 13 iron ore samples investigated as oxygen carriers (Figure S1) (PDF)
