# Supplementary material for: Optimization, Characterization, and Selection of Iron Ores as Oxygen Carriers for Application in Chemical Looping Processes
Source: ACS Omega. 2026 Jan 22;11(4):4997–5015. doi: 10.1021/acsomega.5c05641 (PMC12878788; doi:10.1021/acsomega.5c05641)
Supplement: Supplementary file 1 [file ao5c05641_si_001.pdf]

Supplementary material

Optimization, characterization and selection of iron ores as oxygen carriers for application in chemical looping processes

Gineide Conceição dos Anjos[a], Tiago Roberto da Costa[a,b], Rebecca Araújo Barros do Nascimento Santiago[a], Gislane Pinho de Oliveira[a,c], Tomaz Rodrigues de Araújo[a], Rodolfo Luiz Bezerra de Araújo Medeiros[a], Ângelo Anderson da Silva de Oliveira[a], Dulce M. A. Melo[a,d], Renata Martins Braga[a,e],

[a]*Environmental Technology Laboratory (LabTam), Federal University of Rio Grande do Norte, 59078-970, Natal, RN, Brazil*

[b]*Federal Institute of Rio Grande do Norte – IFRN, Campus Currais Novos/Brazil.*

[c]*Coordination of Environmental Engineering, Federal University of Maranhão, MA-140, km 04, 65800-000,Balsas, MA, Brazil.*

[d]*Postgraduate Program in Chemistry, Federal University of Rio Grande do Norte, 59078-970, Natal, RN, Brazil*

[e]*Agricultural School of Jundiaí, Federal University of Rio Grande do Norte, 59078-970, Natal, RN, Brazil*

*Corresponding author

Supplementary Discussion for Figure S1

The complete morphological and surface chemical characterization of all thirteen iron ore samples by SEM-EDS is presented in Figure S1. This comprehensive dataset provides a complete overview of the textural and compositional diversity of the investigated materials.

Hematite-based samples (Figures S1a-k): The hematite-based oxygen carriers exhibited typical morphological features of iron ores, including slightly rough textures and irregular, pointed shapes. Surface chemical composition mapping by EDX revealed significant compositional variations among the samples. The FeHV (Figure S1j), FeHL (Figure S1k), and FeHP (Figure S1i) samples showed the highest iron contents in ascending order, corroborating the XRF data. Conversely, the FeLJ (Figure S1h), FeHJ-w (Figure S1e), FeHC (Figure S1a), FeLC (Figure S1c), and FeLC-2 (Figure S1d)

materials presented the highest percentages of silicon in ascending order. The presence of manganese was detected in FeHC (Figure S1a) and FeHP (Figure S1i) samples (%Mn < 6.5%), consistent with XRF and XRD results. Calcium was identified in the chemical mapping of FeHJ (Figure S1f) and FeHL (Figure S1k) samples, while small amounts of titanium (Ti) were detected in FeMC (Figure S1b), FeLC-2 (Figure S1d), and FeHL (Figure S1k) samples.

Ilmenite-based samples (Figures S1l-m): The two ilmenite samples exhibited markedly different morphologies. FeTiHL (Figure S1l), which underwent calcination to remove the organic binder from the granulation process, showed agglomerated particles with rough surfaces and a surface composition of 55.88% Fe, 12.44% Ti, and 30.14% O. This composition suggests iron migration to the particle surface during calcination, forming an iron-rich outer layer.[45] In contrast, FeTiHM (Figure S1m), used in its natural state, displayed dense particles with rounded shapes, no visible porosity, and a surface composition of 24.0% Fe, 30.0% Ti, and 40.7% O, along with trace impurities (Zr, Al, Si, Mn). These morphological and compositional differences reflect the distinct processing histories of the two ilmenite samples and may influence their activation behavior during redox cycling.

The comprehensive SEM-EDS characterization confirms the compositional data obtained by XRF and XRD, demonstrating the heterogeneity of the investigated iron ores and providing insights into the relationship between surface chemistry, morphology, and oxygen carrier performance.

Figure S1. Complete SEM-EDS Characterization

Figure S1. Comprehensive scanning electron microscopy (SEM) imaging and energy dispersive X-ray spectroscopy (EDX) chemical mapping of all thirteen iron ore samples investigated as oxygen carriers: (a) FeHC, (b) FeMC, (c) FeLC, (d) FeLC-2, (e) FeHJ-w, (f) FeHJ, (g) FeHJ-2, (h) FeLJ, (i) FeHP, (j) FeHV, (k) FeHL, (l) FeTiHL, and (m) FeTiHM. The main manuscript (Figure 5) presents selected high-performance samples for detailed discussion.

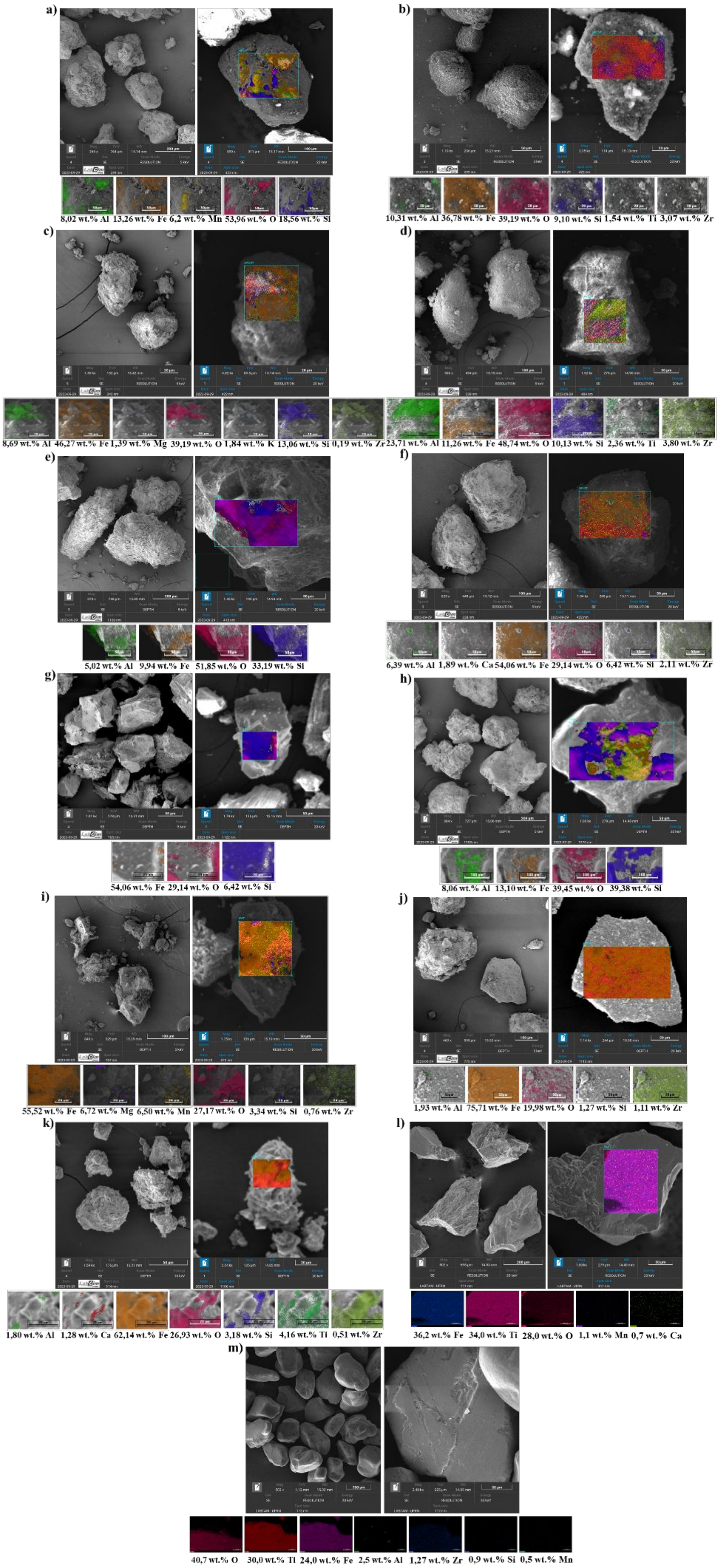
a)
8,02 wt.% Al 13,26 wt.% Fe 6,2 wt.% Mn 53,96 wt.% O 18,56 wt.% Si
b)
10,31 wt.% Al 36,78 wt.% Fe 39,19 wt.% O 9,10 wt.% Si 1,54 wt.% Ti 3,07 wt.% Zr
c)
8,69 wt.% Al 46,27 wt.% Fe 1,39 wt.% Mg 39,19 wt.% O 1,84 wt.% K 13,06 wt.% Si 0,19 wt.% Zr
d)
23,71 wt.% Al 11,26 wt.% Fe 48,74 wt.% O 10,13 wt.% Si 2,36 wt.% Ti 3,80 wt.% Zr
e)
5,02 wt.% Al 9,94 wt.% Fe 51,85 wt.% O 33,19 wt.% Si
f)
6,39 wt.% Al 1,89 wt.% Ca 54,06 wt.% Fe 29,14 wt.% O 6,42 wt.% Si 2,11 wt.% Zr
g)
54,06 wt.% Fe 29,14 wt.% O 6,42 wt.% Si
h)
8,06 wt.% Al 13,10 wt.% Fe 39,45 wt.% O 39,38 wt.% Si
i)
55,52 wt.% Fe 6,72 wt.% Mg 6,50 wt.% Mn 27,17 wt.% O 3,34 wt.% Si 0,76 wt.% Zr
j)
1,93 wt.% Al 75,71 wt.% Fe 19,98 wt.% O 1,27 wt.% Si 1,11 wt.% Zr
k)
1,80 wt.% Al 1,28 wt.% Ca 62,14 wt.% Fe 26,93 wt.% O 3,18 wt.% Si 4,16 wt.% Ti 0,51 wt.% Zr
l)
36,2 wt.% Fe 34,0 wt.% Ti 28,0 wt.% O 1,1 wt.% Mn 0,7 wt.% Ca
m)
40,7 wt.% O 30,0 wt.% Ti 24,0 wt.% Fe 2,5 wt.% Al 1,27 wt.% Zr 0,9 wt.% Si 0,5 wt.% Mn
